# *HRAS* germline mutations impair LKB1/AMPK signaling and mitochondrial homeostasis in Costello syndrome models

**DOI:** 10.1172/JCI131053

**Published:** 2022-04-15

**Authors:** Laetitia Dard, Christophe Hubert, Pauline Esteves, Wendy Blanchard, Ghina Bou About, Lyla Baldasseroni, Elodie Dumon, Chloe Angelini, Mégane Delourme, Véronique Guyonnet-Dupérat, Stéphane Claverol, Laura Fontenille, Karima Kissa, Pierre-Emmanuel Séguéla, Jean-Benoît Thambo, Lévy Nicolas, Yann Herault, Nadège Bellance, Nivea Dias Amoedo, Frédérique Magdinier, Tania Sorg, Didier Lacombe, Rodrigue Rossignol

**Affiliations:** 1 INSERM U1211, Bordeaux, France.; 2 Bordeaux University, Bordeaux, France.; 3 CELLOMET, Functional Genomics Center, Bordeaux, France.; 4 Université de Strasbourg, CNRS, INSERM, CELPHEDIA, PHENOMIN, Institut Clinique de la Souris, Illkirch, France.; 5 Aix-Marseille University, INSERM, Marseille Medical Genetics, Marseille, France.; 6 Medical Genetics Department, Bordeaux University Hospital, Bordeaux, France.; 7 Vect’UB, vectorology platform, INSERM US005 — CNRS UMS 3427-TBM-Core, and; 8 Plateforme Proteome, University of Bordeaux, Bordeaux, France.; 9 AZELEAD, Montpellier, France.; 10 CHU Bordeaux, Haut-Lévèque Hospital, Cardiology Department, Bordeaux, France.; 11 IHU LYRIC, Pessac, France.; 12 Université de Strasbourg, CNRS, INSERM Institut de Génétique et de Biologie Moléculaire et Cellulaire, Illkirch, France.

**Keywords:** Metabolism, Bioenergetics

## Abstract

Germline mutations that activate genes in the canonical RAS/MAPK signaling pathway are responsible for rare human developmental disorders known as RASopathies. Here, we analyzed the molecular determinants of Costello syndrome (CS) using a mouse model expressing HRAS p.G12S, patient skin fibroblasts, hiPSC-derived human cardiomyocytes, a HRAS p.G12V zebrafish model, and human fibroblasts expressing lentiviral constructs carrying HRAS p.G12S or HRAS p.G12A mutations. The findings revealed alteration of mitochondrial proteostasis and defective oxidative phosphorylation in the heart and skeletal muscle of CS mice that were also found in the cell models of the disease. The underpinning mechanisms involved the inhibition of the AMPK signaling pathway by mutant forms of HRAS, leading to alteration of mitochondrial proteostasis and bioenergetics. Pharmacological activation of mitochondrial bioenergetics and quality control restored organelle function in HRAS p.G12A and p.G12S cell models, reduced left ventricle hypertrophy in CS mice, and diminished the occurrence of developmental defects in the CS zebrafish model. Collectively, these findings highlight the importance of mitochondrial proteostasis and bioenergetics in the pathophysiology of RASopathies and suggest that patients with CS may benefit from treatment with mitochondrial modulators.

## Graphical abstract

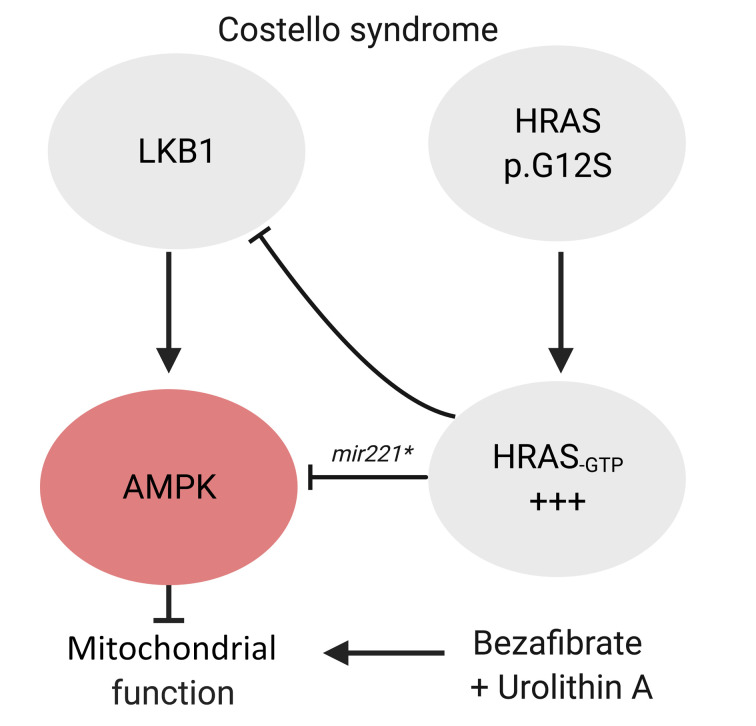

## Introduction

Germline mutations that activate RAS/MAPK signaling are responsible for the “RASopathies,” a group of rare human developmental diseases that affect more than 400,000 individuals in the United States alone. Costello syndrome (CS) has been described as a multiple congenital anomaly syndrome caused by heterozygous activating germline mutations in *HRAS* (1). Most individuals affected by CS carry a mutation in HRAS at the G12 position, and more than 80% of individuals with CS have a p.G12S substitution. Most children with CS exhibit an increased birth weight, dysmorphic craniofacial features, failure to thrive, and gastroesophageal reflux with oral aversion, especially during the newborn period. CS can also involve the skin, with excessive wrinkling and redundancy over the dorsum of the hands and feet, along with deep plantar and palmar creases, as well as an increased risk of developing benign or malignant tumors.

A central feature of CS and other RASopathies is hypertrophic cardiomyopathy (HCM; refs. 2, 3). Musculoskeletal abnormalities such as hypotonia have also been reported in CS (4). Furthermore, HCM was observed in transgenic mutant *Hras* mouse models and in patients expressing constitutively active forms of HRAS (5–10); however, the molecular mechanisms linking HRAS activation with cardiac dysfunction remain unknown. However, cardiac involvement remains a major determinant for the prognosis of CS, raising the need to identify the molecular causes of HCM and propose adapted therapeutic strategies. Several lines of evidence have indicated that HCM typically associates with energetic insufficiency caused by bioenergetic alterations of the heart (11–13). According to the American Heart Association, “alterations in mitochondrial function are increasingly being recognized as a contributing factor in myocardial infarction and in patients presenting with cardiomyopathy” (14). Therefore, we hypothesized that CS could include early alterations of heart bioenergetics participating in the development of HCM. Our hypothesis was also based on a prospective screening of 18 patients with various RAS/MAPK pathway defects that detected biochemical signs of disturbed oxidative phosphorylation (OXPHOS) (15) as well as on the partial overlap of clinical manifestations between mitochondrial diseases and RASopathies (16). The central role of mitochondrial dysfunction in HCM pathophysiology was also demonstrated in vivo using transgenic mice, with the following KOs: *Prkaa2* (17), *Ppargc1a* (18, 19), *Nfe2l2* (20), *Esrra* (21), *Ppara* (22), *Clpp* (23) or *Yme1l1* (24).

In the present study, we explored the bioenergetics of the heart and skeletal muscle in different cell and animal models of CS and discovered alteration in the molecular control of mitochondrial proteostasis. We tested the effect of mitochondrial turnover and bioenergetics pharmacological stimulation on HCM prevention at the preclinical stage in the CS mouse and zebrafish models.

## Results

### Patients with CS and a mouse model exhibit left ventricular HCM.

**Figure 1:**
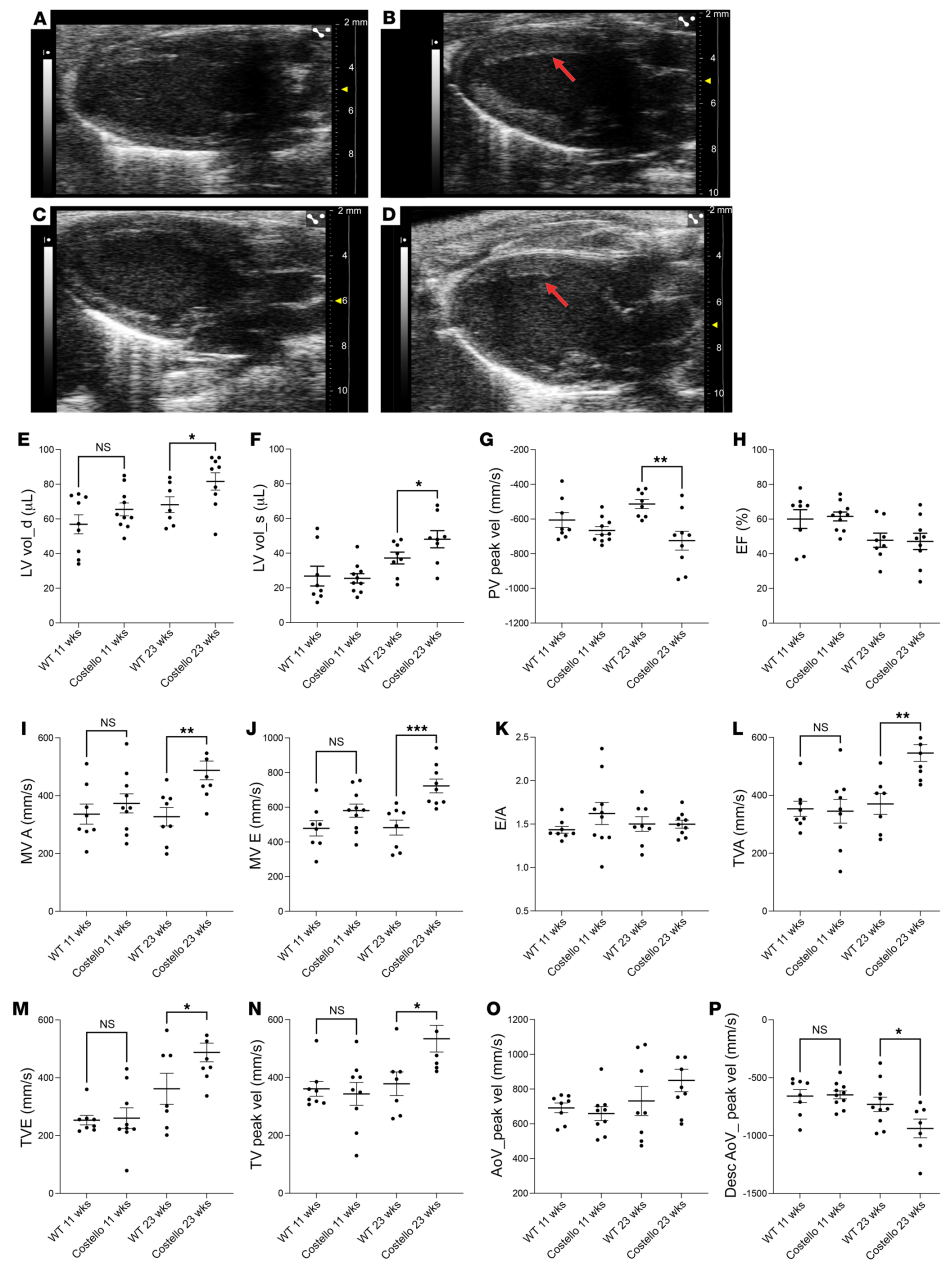
Hypertrophic cardiomyopathy in patients with CS and the mouse model. Parasternal echography was performed in (**A** and **C**) WT (HRAS) or (**B** and **D**) Costello (HRAS p.G12S) mice at 11 weeks (**A** and **B**) and 23 weeks (**C** and **D**) of age. Left ventricle hypertrophy is indicated by arrows. (**E**) Left ventricle volume at diastole (LV vol_d), (**F**) left ventricle volume at systole (LV vol_s), (**G**) pulmonary valve peak velocity (PV peak vel), (**H**) ejection fraction (EF), (**I**) mitral valve flow A wave maximal velocity (MV A), (**J**) mitral valve flow E wave maximal velocity (MV E), (**K**) E/A ratio, (**L**) tricuspid valve flow A wave maximal velocity (TVA), (**M**) tricuspid valve flow E wave maximal velocity (TVE), (**N**) tricuspid valve peak velocity (TV peak vel), (**O**) aorta peak velocity (AoV_peak vel), and (**P**) descending aorta peak velocity (Desc AoV_peak vel). The data are expressed as the mean ± SEM. *P < 0.05, **P < 0.01, ***P < 0.001 (unpaired *t* test was performed between the CS mouse group and the corresponding age-matched WT animals). HCM, hypertrophic cardiomyopathy.

First, we regrouped a cohort of 10 patients with CS (Supplemental Table 1; supplemental material available online with this article; https://doi.org/10.1172/JCI131053DS1) to investigate heart structure and function. Echocardiography revealed a characteristic left ventricular hypertrophy in 70% of the patients (Supplemental Figure 1, A–D). Electrocardiograms obtained of patients with CS further revealed that 50% of the patients had abnormal deviation of the heart axis (Supplemental Table 2). Two patients had a right deviation of the electrical axis with discrete echocardiographic left ventricular hypertrophy. Furthermore, pathologic Q waves were present in 40% of patients, and all patients with pathologic Q waves had only discrete ventricular hypertrophy on echocardiography. In the heterozygous HRAS p.G12S mouse model of CS, we observed increased left ventricle volume (Figure 1, A–F, and Supplemental Figure 1, F and G) and cardiomegaly (Supplemental Figure 1E and Supplemental Figure 2). A significant increase in systolic arterial blood pressure was also observed in these animals at 8 and 20 weeks of age (Supplemental Figure 1H) without alteration in heart beating rate (Supplemental Figure 1I). Likewise, hypertension was observed in all the patients with CS considered in this study (Supplemental Table 1). Further evaluation of the heart structure and function by echocardiography confirmed the increase in left ventricle volume at diastole and systole (Figure 1, E and F). Evaluation of the left ventricular diastolic function, including early and late mitral flow E wave velocities revealed a significant increase in these 2 parameters in 23-week-old CS mice (Figure 1, I–K). These findings might reflect increased left atrial filling pressures in the left atrium. The E wave velocity through the tricuspid valve was also increased (Figure 1, L–N), suggesting occurrence of tricuspid regurgitation. Tricuspid regurgitation, in combination with left ventricular dysfunction was associated with excess mortality in previous studies (25). Last, the pulmonary vein peak velocity was also reduced in 23-week-old CS mice (Figure 1G). Comparison of the echocardiography studies performed at 11 and 23 weeks of age showed that the cardiac disease was progressive and did not reach yet the state of heart failure, as the ejection fraction and the E/A ratio remained unaltered (Figure 1, H and K, and Supplemental Figure 3). Nevertheless, the descending aorta peak velocity was significantly reduced in the CS mouse heart (Figure 1, O and P), and the establishment of a ventricular dilation (Figure 1, A and F) with altered mitral and tricuspid flow E wave velocities (Figure 1, I, J, and L–N) was significant in the CS mouse model. These observations indicate that left ventricular cardiac hypertrophy is a characteristic feature of CS in most patients and in the HRAS p.G12S mouse model.

### The CS mouse model exhibits heart and skeletal muscle bioenergetic dysfunction.

**Figure 2:**
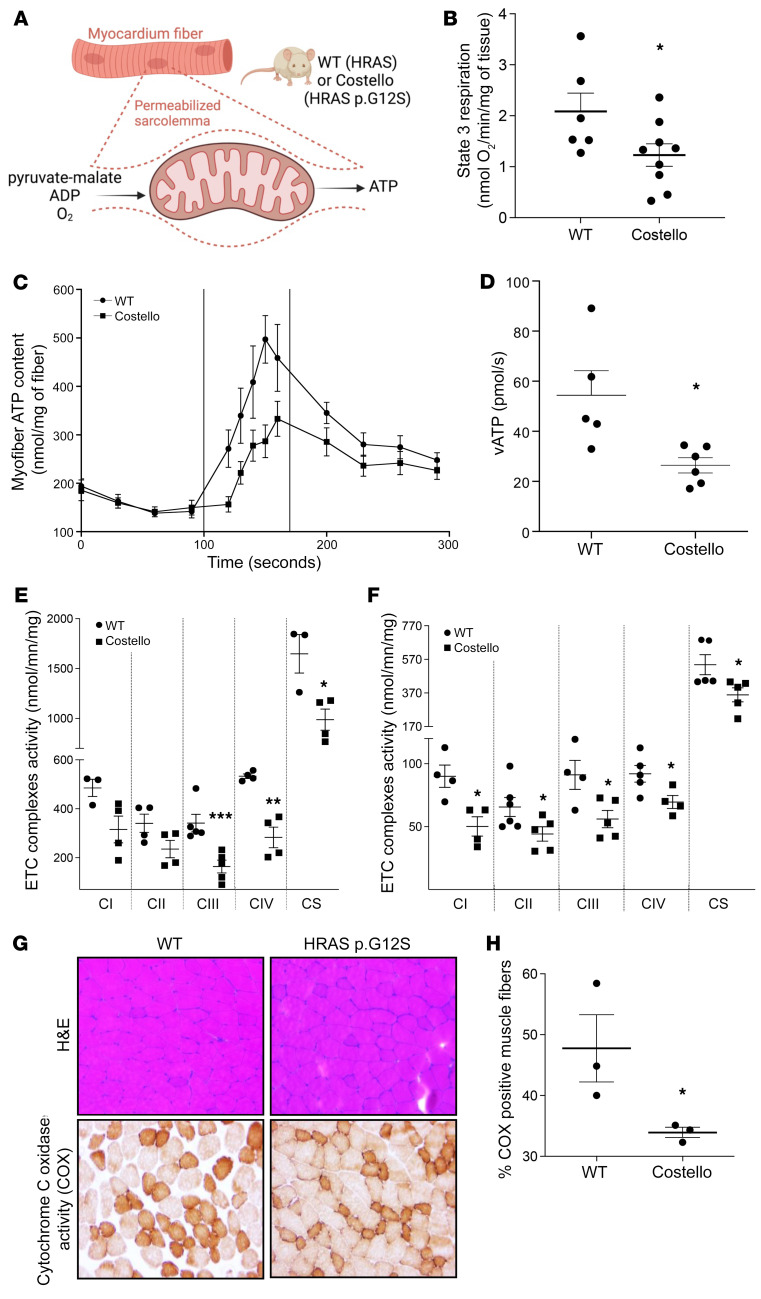
Mitochondrial bioenergetics is altered in situ in the CS mouse heart and skeletal muscle. (**A**) Permeabilized heart muscle fibers bioenergetics evaluation methods. (**B**) Rate of coupled (ADP-stimulated “state 3”) respiration was determined in situ using high-resolution respirometry (WT, *n =* 6; Costello, *n =* 9). (**C** and **D**) Rate of mitochondrial ATP synthesis (vATp) determined in heart-permeabilized muscle fibers (WT, *n =* 5; Costello, *n =* 6). (**E**) Electron transport chain (ETC) complex enzymatic activities determined in WT or Costello mouse model hearts (WT, *n =* 4; Costello, *n =* 4). (**F**) Respiratory chain complex enzymatic activities determined in WT or Costello mouse model skeletal muscle (WT, *n =* 4; Costello, *n =* 4). (**G**) Histo-enzymology staining of the respiratory chain complex IV (COX) specific activity. Muscle fibers and their nuclei were stained using H&E. Original magnification, ×1000. (**H**) Quantification of the COX-positive muscle fibers (WT, *n =* 3; Costello, *n =* 3). Data are expressed as the mean ± SEM. *P < 0.05, **P < 0.01, ***P < 0.001 (unpaired *t* test).

Mitochondrial dysfunction is an important determinant of HCM (26); thus, we investigated heart bioenergetics in the CS mouse model. The in situ evaluation of OXPHOS (Figure 2A) was performed in heart-permeabilized muscle fibers obtained from control or heterozygous 12-week-old CS mice. This analysis revealed a reduced rate of coupled respiration (state 3) associated with a decreased rate of ATP synthesis (Figure 2, B–D). Measurement of the respiratory chain complex enzymatic activity (Figure 2, E and F) demonstrated a generalized decrease in complex I–IV activity in the CS mouse heart (Figure 2E). Respiratory chain complex enzymatic activity defects were also discovered in the skeletal muscle of CS mice (Figure 2F). Furthermore, histo-enzymology determination of complex IV activity in the skeletal muscle showed a strong reduction in the number of COX-positive fibers (Figure 2, G and H). Accordingly, a reduction of state 3 respiration and mitochondrial ATP synthesis were observed in permeabilized skeletal muscle fibers obtained from 12-week-old heterozygous CS mice (Supplemental Figure 3). These findings revealed the existence of mitochondrial dysfunction in both the myocardium and skeletal muscle of CS mice.

### Mitochondrial proteostasis is altered in CS.

**Figure 3:**
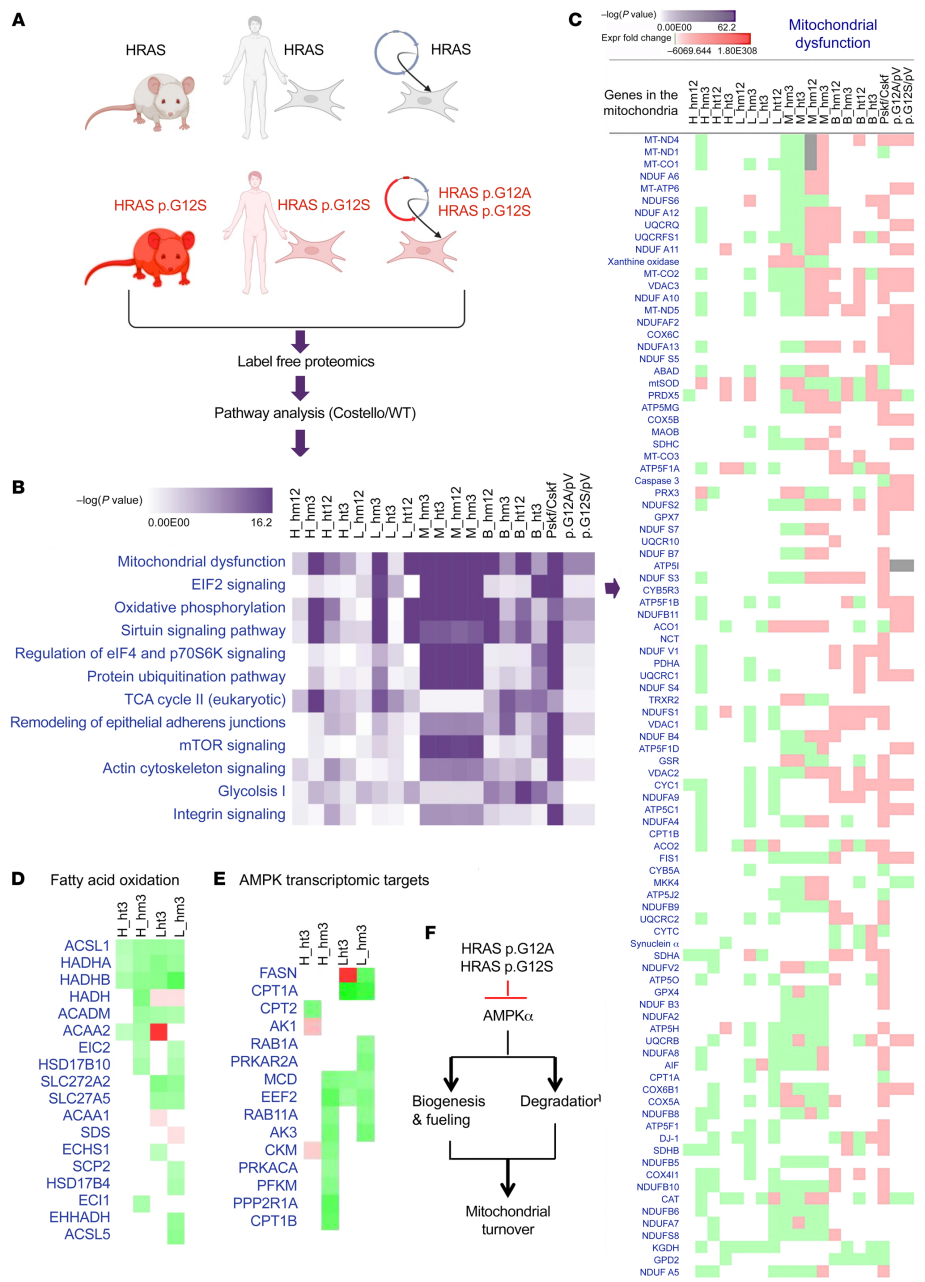
Mitochondrial proteostasis is compromised in Costello syndrome. (**A**) Description of the 3 types of Costello syndrome (CS) models used in the label-free proteomic analysis. A differential proteome was obtained between WT and CS models. Ingenuity Pathway Analysis (Qiagen; version 01-07) was used to perform a comparative analysis of the data. *n =* 3 was used for each sample. (**B**) Top canonical pathways altered in the CS biological models [ranked by –log(*P* value)]. (**C**) Detail of the changes in the “mitochondrial dysfunction” category (expression fold change; green, downregulation; red, upregulation). (**D** and **E**) Changes in the fatty acid oxidation and AMPK signaling (indirect transcriptomic targets) proteins detected in the hearts and livers of 3-week-old CS mice (heterozygous or homozygous HRAS p.G12S) compared with WT mice. (**F**) Working hypothesis on the effect of mutant HRAS p.G12A and HRAS p.G12S on mitochondrial biogenesis and degradation via inhibition of AMPK signaling. H, heart; B, brain; L, liver; M, skeletal muscle; ht, heterozygous; hm, homozygous; 3, 3-week-old mice; 12, 12-week-old mice; Pskf, patient skin fibroblasts; Cskf, control skin fibroblasts; p.G12A, human skin fibroblasts transduced with HRAS p.G12A–expressing lentiviral plasmid; p.G12S, human skin fibroblasts transduced with HRAS p.G12S–expressing lentiviral plasmid; pV, human skin fibroblasts transduced with empty lentiviral plasmid.

To unravel the molecular mechanisms underpinning early OXPHOS dysfunction in the CS mouse model we performed a label-free proteomic analysis (Figure 3A) of (a) 4 CS mouse tissues (skeletal muscle, heart, liver, and brain) obtained at 3 and 12 weeks of age from homo- and heterozygous HRAS p.G12S animals, (b) human primary skin fibroblasts obtained from 3 patients with CS carrying HRAS p.G12S or HRAS p.G12A mutations, and (c) human primary skin fibroblasts expressing ectopic HRAS p.G12S or HRAS p.G12A lentiviral constructs (*n =* 4 clones for each construct). Comprehensive analysis of the cellular biochemical processes altered by mutant HRAS (Figure 3B) consistently identified “mitochondrial dysfunction” and “OXPHOS” with the highest score (–log(*P* value)) in each of the CS models, suggesting a generalized alteration of the mitochondrial proteome homeostasis consecutive to germline and somatic HRAS mutation. However, the direction of the changes observed in the content of selected mitochondrial proteins varied between tissues. For instance, a predominant decrease of OXPHOS components was detected in the heart and liver, while a dominating accumulation of these proteins was observed in the muscle, brain, and skin fibroblasts from patients with CS as well as in the related mutated HRAS p.G12S– and p.G12A–expressing cell models (Figure 3C). A focus on the common molecular changes that occurred in the hearts and livers, 2 tissues with very different functions, of 3-week-old heterozygous and homozygous CS mice revealed a reduction of fatty acid oxidation enzymes and 5′ AMP-activated protein kinase (AMPK) indirect transcriptomic targets (Figure 3, D and E). These findings raised the hypothesis that HRAS p.G12S mutation could trigger alteration of mitochondrial proteome homeostasis with a more pronounced effect on mitochondrial biogenesis or degradation according to the tissue considered and stage of development. Given the role of the LKB1/AMPK signaling axis in the tissue-specific control of mitochondrial biogenesis and degradation, we hypothesized that altered LKB1 and/or AMPK function could be a consequence of HRAS hyperactivation in CS (Figure 3F).

### HRAS hyperactivation inhibits the LKB1/AMPK pathway.

**Figure 4:**
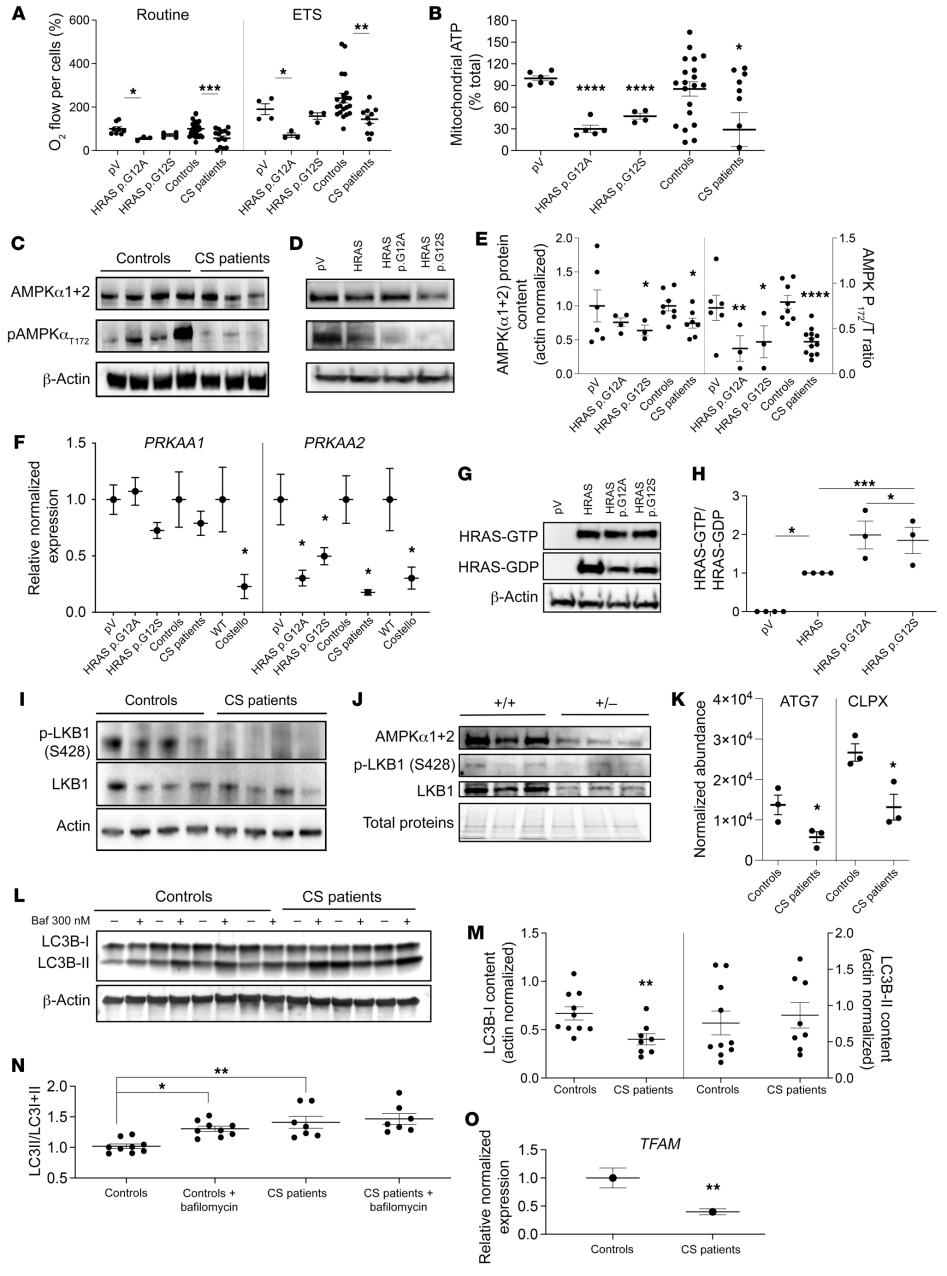
AMPKα2 expression is inhibited by mutant HRAS. (**A**) Mitochondrial respiration determined in skin fibroblasts from patients with CS and in WT human fibroblasts transduced with an empty plasmid (pV) or mutant forms of HRAS (*n ≥* 3 for each condition). (**B**) Mitochondrial ATP content (expressed as a percentage of the total ATP) (*n ≥* 3). (**C**) Total AMPK α subunits (α1+α2) and T172_phospho-AMPK protein content were quantified in cells from patients with CS (*n =* 6) and WT controls (*n =* 6). (Phospho-AMPK/[phospho-AMPK+total AMPK]) was denominated “AMPK P_172_/T_ratio.” (**D** and **E**) AMPK and T172_phospho-AMPK protein content determined in mutant HRAS-expressing human skin fibroblasts (pV, empty plasmid; HRAS, WT gene; HRAS p.G12A and HRAS p.G12S, mutated forms of the gene) (*n ≥* 3). (**F**) mRNA content of 2 AMPK subunits (α1 and α2) in different CS models: CS mouse heart, fibroblasts from patients with CS, and mutant HRAS cell models (*n =* 3). (**G** and **H**) Effect of HRAS mutations on HRAS activity (*n =* 3). (**I**) LKB1 expression level and its S428 phosphorylation status on control fibroblasts (*n =* 7) and fibroblasts of patients with CS (*n =* 6). (**J**) Protein content of AMPKα1+α2, T172_phospho-AMPK, LKB1, and S428_P-LKB1 in heart samples from Costello (HRAs p.G12S) or WT (HRAS) mice. (**K**) Protein levels of ATG7 and CLPX determined by mass spectrometry in cells from patients with CS (*n =* 3) as compared with control cells (*n =* 3). (**L** and **M**) LC3B-I and LC3B-II levels were determined in skin fibroblasts from controls or patients in presence or absence of 300 nM of bafilomycin A1 (*n =* 3). (**N**) LC3 activation level was expressed as (LC3-II/[LC3I+LC3II]). (**O**) Relative normalized expression of TFAM mRNA in cells from patients with CS (*n =* 3). Data are expressed as the mean ± SEM. One-way ANOVA with Dunnett’s correction for multiple testing was used to compare the 3 groups of cells expressing HRAS p.G12A or HRAS p.G12S with the empty plasmid control, while a *t* test was used to compare the 2 groups of cells obtained from patients with CS and controls or the 2 groups of mice (Costello or WT). *P < 0.05, **P < 0.01, ***P < 0.001, ****P < 0.0001.

Investigation of the bioenergetic changes in skin fibroblasts from patients with CS and ectopic HRAS p.G12A or HRAS p.G12S-expressing human skin fibroblasts revealed a reduction of OXPHOS function and capacity (Figure 4, A and B) associated with alteration of respiratory chain proteostasis (Figure 3, B and C). In line with the hypothesis of a deregulated LKB1/AMPK signaling axis, we first determined the global content of AMPKα1 and α2 subunits by Western blot using a polyclonal antibody that could not distinguish between the 2 subunits (Figure 4, C–E). The results showed a global decrease in AMPKα1+α2 subunits in CS cell models, cells from patients with CS, and human skin fibroblasts expressing mutant HRAS p.G12A or HRAS p.G12S. To add precision to our findings, we performed Q-PCR investigations to detect the specific changes in PRKAA1 or PRKAA2 subunits (Figure 4F). The results showed a significant downregulation (–75%) of the PRKAA2 subunit mRNA content in cells from patients with CS and mutant HRAS p.G12A and p.G12S cell models but also 12-week-old HRAS p.G12S heterozygous CS mouse hearts. As AMPKα2 mediates AMPK activation via threonine 172 phosphorylation, a corresponding reduced expression of the pAMPK (thr172) content was detected in the different CS cell models (Figure 4, C–E). Our findings demonstrated that mutant HRAS p.G12A and p.G12S stimulates HRAS activation (Figure 4, G and H) and inhibits, in turn, the expression of the AMPKα2 subunit that is essential for AMPK activation. Moreover, analysis of the LKB1 phospho-activation status, as determined by the phosphorylation level of serine 428, revealed a strong reduction in cells from patients with CS. Finally, the phosphorylation level of ACC, a canonical target of AMPK was reduced in patient skin fibroblasts, although the level of ACC phosphorylation was low in our experimental conditions (Supplemental Figure 4). Therefore, the LKB1/AMPK signaling axis was inhibited at 2 levels in fibroblasts from patients with CS (Figure 4I). Similar findings were obtained in 3-week-old CS mouse hearts, as the AMPKα1+α2 and LKB1 protein content were substantially decreased (Figure 4J). In cells from patients with CS, the observed accumulation of mitochondrial proteins (Figure 3C) suggested a predominant alteration of mitochondrial proteostasis at the level of mitochondrial degradation, a complex process dependent on autophagy-mediated bulk degradation of mitochondria (mitophagy), and protease-mediated specific degradation of mitochondrial proteins. Accordingly, comparative proteomics revealed a marked reduction of the autophagy regulator ATG7 and of the mitochondrial protease CLPx (Figure 4K), 2 elements essential for mitochondrial clearance. Further evaluation of the mechanisms involved in mitochondrial proteostasis was performed in the CS mouse heart (Supplemental Figure 5) and skin fibroblasts from patients with CS (Supplemental Figure 6). This analysis revealed that AMPK, CRIF1, and SQSTM1/p62 were consistently reduced both in CS mouse heart and in skin fibroblasts from patients with CS. Previous work has already shown that AMPK, CLPX, ATG7, CRIF1, and SQSTM1/p62 inhibition trigger defective mitochondrial proteostasis, cellular bioenergetics impairment, and inhibition of the autophagic flux (27–30). Accordingly we measured a defective mitochondrial bioenergetics (Figure 2, B–D) and decreased autophagic flux (Figure 4, L–N) in the different CS cell models. The mitochondrial proteostasis regulator CLPX, which was downregulated in CS cells, had previously been identified as a transcriptomic target of AMPK and PGC1α (31, 32), 2 large-scale transcriptional regulators strongly downregulated in CS cells.

Mitochondrial biogenesis, the second arm of mitochondrial turnover controlled by AMPK, was evaluated by measuring the expression level of TFAM in cells from patients with CS using Q-PCR (Figure 4O). TFAM content was decreased in cells from patients with CS (as well as in CS mouse heart; Supplemental Figure 5), in line with an alteration of mitochondrial biogenesis. These findings indicate that the LKB1/AMPK signaling axis is altered in cells from patients with CS and CS mouse hearts and that mitochondrial biogenesis and degradation processes are also impaired in these cells. However, the proteomic investigation of CS mouse hearts revealed additional changes in proteins specifically involved in the pathophysiology of cardiomyopathy (Supplemental Figure 7), such as CRYAB, TNN or MYBPC3, suggesting that LKB1/AMPK signaling deregulation is only one mechanism contributing to the complexity of CS heart pathophysiology, as expected in a condition of HRAS/MAPK pathway overactivation. Moreover, comparative analysis of 4 CS mouse tissues taken at 3 weeks of age suggested that alteration of mitochondrial proteostasis in CS mice is tissue specific (Supplemental Figure 8).

### miR-221* inhibits AMPKα2 expression in mutant HRAS-expressing cells.

**Figure 5:**
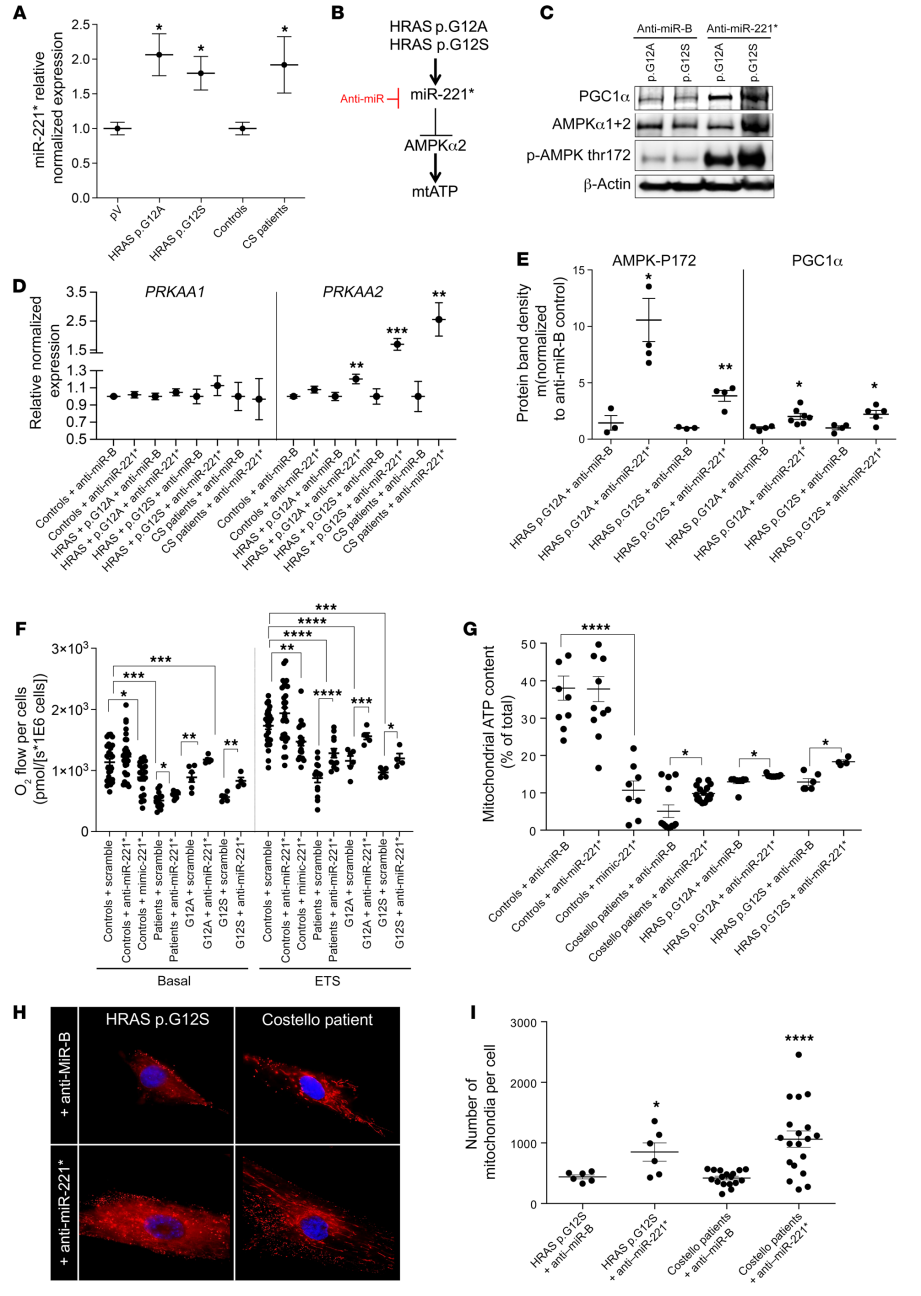
miR-221-5p inhibits AMPKα2 expression in an HRAS-dependent manner. (**A**) miR-221-5p (miR-221*) expression in skin fibroblasts from patients with CS and related transgenic cell models (*n =* 3). (**B**) Description of the HRAS/miR-221*/AMPK pathway. (**C** and **D**) Rescue of AMPKα2 expression by anti–miR-221-5p at the protein and the mRNA levels, respectively (*n =* 3). (**E**) Rescue of AMPKα2 expression, T172_P-AMPK/total AMPK ratio, and PGC1α expression by the anti–miR-221-5p (*n ≥* 3). (**F**) Rescue of mitochondrial respiration by anti–miR-221-5p and its inhibition by a miR-221-5p mimic (*n ≥* 3). (**G**) Rescue of mitochondrial steady-state ATP content by the anti–miR-221-5p (*n ≥* 3). (**H** and **I**) Rescue of mitochondrial particles number by anti–miR-221-5p (*n =* 3). Original magnification, ×1000. Two-way ANOVA with Dunnett’s correction for multiple testing was used to compare the 3 groups of cells expressing HRAS p.G12A, HRAS p.G12S, or the empty plasmid control (pV) treated with the anti-miR scramble or the anti–miR-221-5p. Unpaired *t* test was used to compare the 2 groups of cells obtained from patients with CS and controls. Data are expressed as the mean ± SEM.*P < 0.05, **P < 0.01, ***P < 0.001, ****P < 0.0001.

Parcellar evidence suggested the existence of a RAS/miR-221*/AMPK regulatory axis in human cancer cells (33–36). Moreover, miR-221* overexpression was observed in patients with cardiac hypertrophy after sudden death (37), as can occur in patients with CS. Thus, we evaluated the implication of this pathway in CS pathophysiology using different approaches. First, in silico analysis of the differential proteomics data obtained in skin fibroblasts from patients with CS and in mutant HRAS-expressing cells showed that a common core of 71 proteins downregulated by germline *HRAS* mutation were predicted targets of miR-221* (Supplemental Figure 9, A and B). These proteins play a role in cellular functions, such as cell migration, cell proliferation, and cell adhesion (Supplemental Figure 9, C and D). Moreover, miR-221* expression was increased in fibroblasts from patients with CS and in human fibroblasts expressing ectopic mutant forms of *HRAS* (Figure 5A), in agreement with our hypothesis (Figure 5B). Furthermore, inhibition of miR-221* expression using a specific anti-miR rescued the expression of AMPKα2 protein, mRNA, and activity in HRAS p.G12S– or HRAS p.G12A–expressing cells (Figure 5, C–E). The rescuing effect of anti–miR-221* was also found at the level of AMPK and PGC1α expression, mitochondrial respiration, and mitochondrial ATP levels in both cells from patients with CS and mutant *HRAS* cell models (Figure 5, E–G). A different sensitivity to the anti–miR-221* was observed between the HRAS p.G12S– and the HRAS p.G12A–expressing fibroblasts (Figure 5C). In the former cell line the increase in total AMPK content was strong, while in the latter it was weak. Still, in the 2 cell lines expressing mutant forms of *HRAS*, the anti–miR-221* triggered a marked increase in the _thr172_P-AMPK/total AMPK ratio. In contrast, the miR-221*-mimic altered cell respiration in control fibroblasts. The number of mitochondrial particles was also increased in cells from patients with CS and in mutant *HRAS* cell models treated with the anti–miR-221* (Figure 5, H and I). Finally, anti–miR-221* stimulated cell growth in glucose-deprived media that forced cells to rely on OXPHOS (38) for survival (Supplemental Figure 10). Taken together, these findings indicate that HRAS p.G12A and p.G12S activate the expression of miR-221* but also that miR-221* inhibits the expression and function of AMPKα2 in skin fibroblasts derived from patients with CS and related cellular models. Our findings also demonstrate that both AMPK phosphorylation status (thr172) and mitochondrial biogenesis can be rescued in vitro using an anti–miR-221*. However, we could not detect the endogenous level of miR-221* in the WT and the CS mouse heart, indicating that inhibition of AMPKα2 by miR-221* may occur by different mechanisms in this tissue. The reduced expression of LKB1 found in the CS mouse heart (Figure 4J), as also observed in skin fibroblasts from patients with CS (Figure 4I), provides an additional mechanism responsible for the inhibition of LKB1/AMPK signaling in CS.

### Human induced pluripotent stem cell–derived CS cardiomyocytes have reduced _ser428_P-LKB1 and AMPKα2 content and bioenergetic impairment.

**Figure 6:**
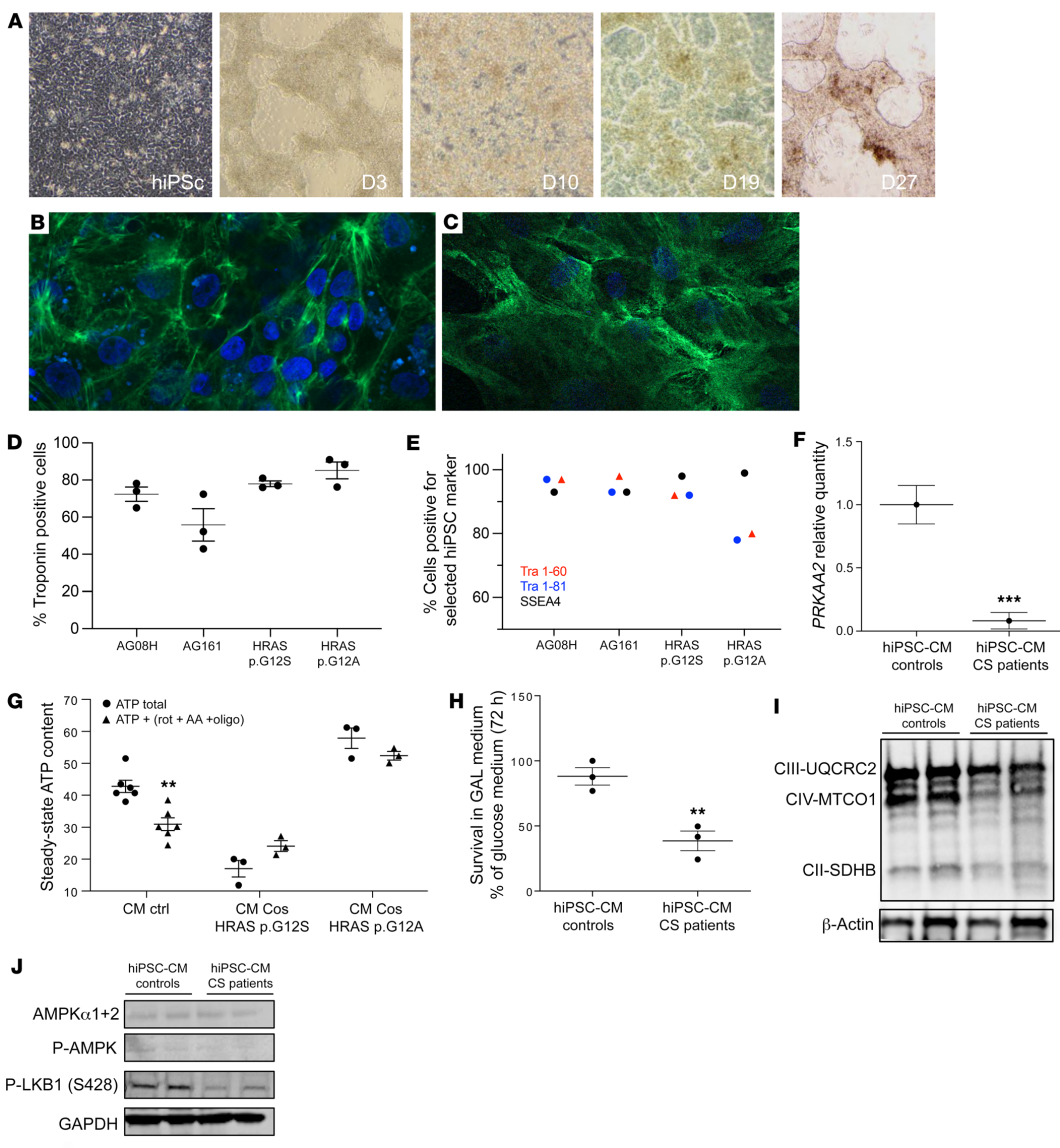
Mitochondrial bioenergetics is impaired in CS hiPSC-derived cardiomyocytes. (**A**) Human induced pluripotent stem cells (hiPSCs) were established by electroporation of different primary fibroblasts cell lines. Characterization of the hiPSC-derived cardiomyocytes using cardiac troponin T staining; 2 examples are shown for (**B**) the control AG08H and (**C**) the Costello G12S hiPSC-derived cardiomyocytes. Original magnification, ×40. (**D**) The percentage of Troponin T–positive cells determined using immunocytochemistry is shown for the 4 lineages of hiPSC-derived cardiomyocytes. (**E**) Expression of the keratin sulfate antigens Tra1-60 and Tra1-81 and the glycolipid antigen SSEA4 was verified by flow cytometry. (**F**) Determination of AMPKα2 (PRKAA2) mRNA expression level by Q-PCR in hiPSC-derived cardiomyocytes, obtained from 2 patients with CS and 2 controls (*n =* 3). (**G**) Measurement of the total cellular steady-state ATP content in hiPSC-derived cardiomyocytes, obtained from 2 patients with CS and 2 controls (*n =* 3). Evaluation of mitochondrial ATP synthesis was performed using inhibitors of oxidative phosphorylation: antimycin A, oligomycin, and rotenone. (**H**) Survival of the hiPSC-derived cardiomyocytes, obtained from 2 patients with CS and 2 controls, in an obligatory oxidative growth medium (*n =* 3). Data are expressed as percentage of the cell number in glucose medium. (**I**) Western blot evaluation of the expression level of various respiratory chain proteins on hiPSC-derived cardiomyocytes, obtained from 2 patients with CS and 2 controls, using the Oxphos kit from Abcam. (**J**) Determination of the protein expression level of AMPK, _T172_-P-AMPK and _ser428_P-LKB1. Protein loading was verified using the GAPDH marker. Data are expressed as the mean ± SEM. Unpaired *t* test was used to compare the 2 groups of hiPSC-CMs (controls and patients). For **G**, 1-way ANOVA with Dunnett’s correction was used to compare the 3 groups of cells. **P < 0.01, ***P < 0.001.

Human induced pluripotent stem cell–derived cardiomyocytes (hiPSC-CMs) were produced from primary skin fibroblasts from 2 patients with CS and from 2 controls (Figure 6A and Supplemental Figure 11). Determination of the percentage of troponin T–positive cells (Figure 6, B and C) at day 30 after differentiation showed 72.55% ± 12% of troponin-positive cells for the AG08H control clone, 55.88% ± 15.6% for the AG161 control clone, 77.89% ± 2.3% for the CS HRAS p.G12A clone, and 86.75% ± 8.7% for the HRAS p.G12S (Figure 6D). Expression of the keratin sulfate antigens Tra1-60 and Tra1-81 and the glycolipid antigen SSEA4 was verified by flow cytometry in the 4 cell lines (Figure 6E). The results showed a high expression (>95%) of these human pluripotent stem cell markers in the different cell lines. Embryoid body formation and characterization were also performed as detailed in Methods (Supplemental Figure 12). Then, we analyzed the markers of defective mitochondrial homeostasis previously identified in CS mouse tissues, skin fibroblasts from patients with CS, and CS cell models. Accordingly, *PRKAA2* expression was strongly reduced in CS hiPSC-CMs (Figure 6F), and mitochondrial ATP synthesis was also impaired (Figure 6G). More precisely, in CS hiPSC-CMs the cellular total ATP level was either not altered by the OXPHOS inhibition (HRAS p.G12A) or even increased by a glycolytic compensatory response (HRAS p.G12S). These data indicate that most of the ATP produced in CS hiPSC-CMs was obtained from glycolysis, as OXPHOS inhibition with oligomycin, antimycin A, and rotenone did not entail a drop in the cellular ATP level. As a result, CS hiPSC-CM viability was reduced in a strictly oxidative cell growth medium (Figure 6H). Finally, we observed a reduction of respiratory chain protein content in these cells (Figure 6I) as well as a decreased _ser428_P-LKB1 content (Figure 6J). No difference in AMPKα1+2 was detected between the CS cardiomyocytes and the WT hiPSC-CMs, and the level of _thr172_P-AMPK was very low in the 2 groups. These findings indicate that the defective mitochondrial bioenergetics phenotype discovered in CS mouse heart and skin fibroblasts from patients with CS was also present in human cardiomyocytes derived from hiPSCs from patients with CS.

### Mitochondrial proteostasis modulators restore organelle homeostasis in CS cell and animal models.

**Figure 7:**
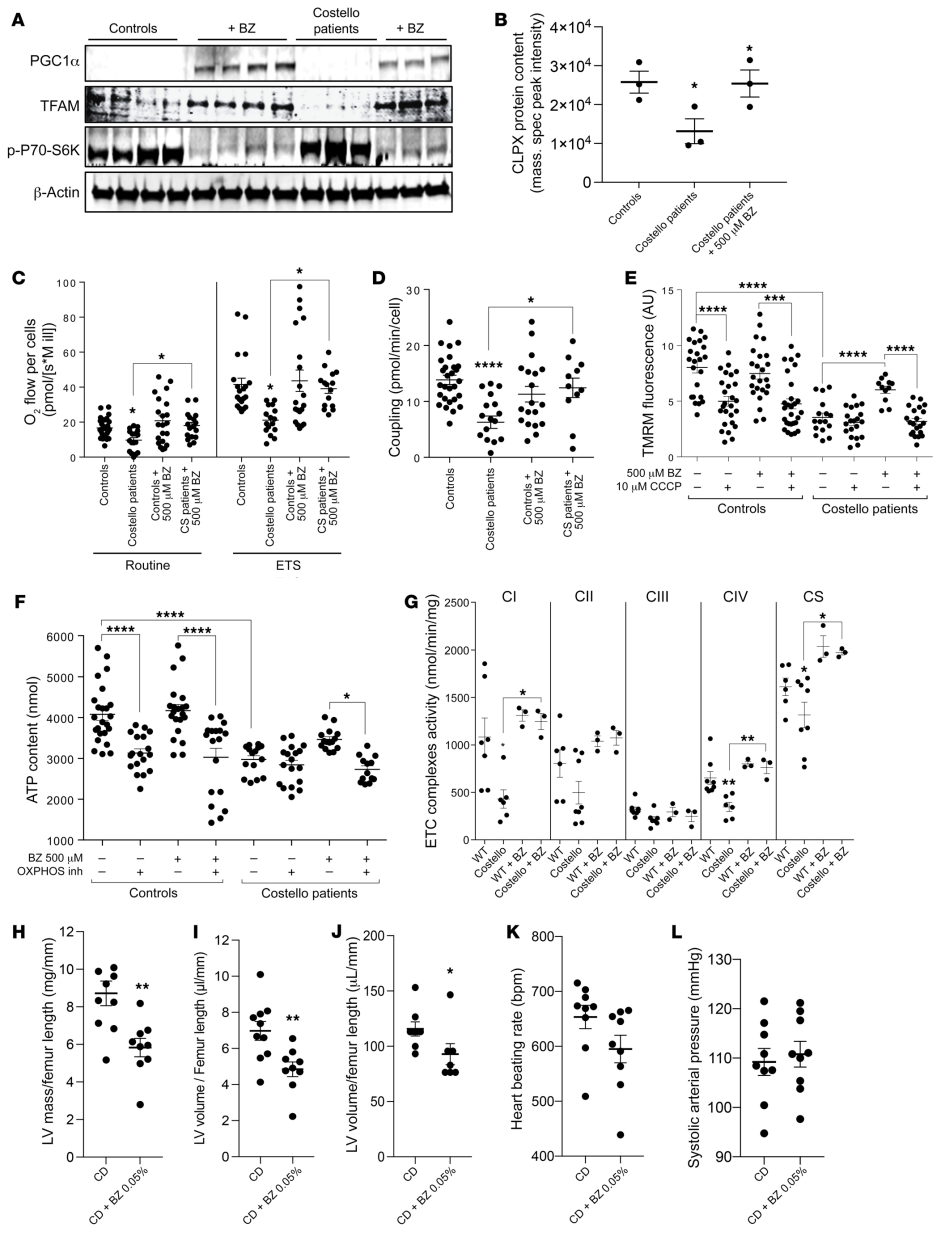
Bezafibrate rescues mitochondrial bioenergetics and prevents left ventricle cardiac hypertrophy in Costello syndrome. (**A**) Increased expression of PGC1α, TFAM, and p-P70S6K by bezafibrate (500 μM; 48 hours) treatment in skin fibroblasts from patients with CS. (**B**) CLPX protein content determined by mass spectrometry of cells from controls, cells from patients with CS, and bezafibrate-treated cells from patients with CS. Quantification was expressed as the normalized peak intensity (*n =* 3). (**C**–**F**) Defective mitochondrial respiration, OXPHOS coupling, mitochondrial transmembrane electric potential, and mitochondrial ATP levels were rescued by bezafibrate (500 μM; 48 hours) in skin fibroblasts from patients with CS (*n =* 3). (**G**) Stimulation of respiratory chain complex enzymatic activity in the hearts of CS mice treated with bezafibrate 0.05% in the diet (for 12 weeks). Effect of the bezafibrate 0.05% in chow diet (CD + BZ 0.05%) on (**H**) left ventricle (LV) mass, (**I**) left ventricle volume, (**J**) heart mass, (**K**) heart beating rate, and (**L**) systolic arterial pressure after 12 weeks of treatment in CS mouse model (*n =* 9) as compared with untreated CS mice fed with chow diet (CD) (*n =* 9). One-way ANOVA with Dunnett’s correction for multiple testing was used to compare the 4 groups of cells (controls treated or untreated with bezafibrate and CS mice treated or untreated with bezafibrate). Unpaired *t* test was used to compare the 2 groups of mice (WT or Costello). *P < 0.05, **P < 0.01, ***P < 0.001, ****P < 0.0001.

The above-described findings suggested the evaluation of a preclinical therapeutic strategy in CS based on the stimulation of mitochondrial protein biogenesis and quality control. Bezafibrate (BZ) was selected to reach this goal, because this drug could stimulate mitochondrial bioenergetics in previous studies (39–44). Accordingly, we observed that BZ increased the expression of the PGC1α 100 KDa form, raised the level of TFAM, and reduced activation of the mTOR pathway (Figure 7A). The enzymatic activity of respiratory chain complexes was also increased by BZ treatment. Proteomics further demonstrated that BZ normalized the level of the autophagy regulator ATG4B but also that of ATG7, ATG3, PARK7, and TOM22 in cells from patients with CS (Supplemental Figure 13). The content of various respiratory chain subunits was also increased by BZ treatment while accumulated subunits were reduced (Supplemental Figure 13). The protein content of mitochondrial protease CLPX was also rescued by the BZ treatment (Figure 7B). The measurement of mitochondrial respiratory fluxes, transmembrane electric potential (ΔΨ), as well as sensitivity to the uncoupler CCCP and OXPHOS coupling efficiency confirmed that 48-hour treatment with 500 μM BZ rescued mitochondrial proteome homeostasis and organelle function in vitro (Figure 7, C–F).

Thus, we performed preclinical treatment of the CS mouse model with BZ 0.05% (Figure 7G). After 12 weeks of treatment with BZ 0.05% in the diet, the enzymatic activity of respiratory chain complex I, complex IV, and citrate synthase was increased (Figure 7G). Moreover, the echocardiography analysis of the treated CS mice revealed that the left ventricle volume was specifically reduced by this treatment (Figure 7, H and I). No significant change was observed in the total mass of the heart (Figure 7J). A substantial reduction of the heart-beating rate was also obtained by the treatment, without modification of the systolic arterial pressure (Figure 7, K and L). Altogether, these findings suggest that the BZ 0.05% treatment prevented development of the left ventricle hypertrophy in the CS mouse model and normalized the heart-beating rate. Still, more specific cardiac functional assessments for reversal/prevention of HCM in CS will need to be performed to prove the therapeutic possibility of using BZ for treating the cardiac disease in CS.

**Figure 8:**
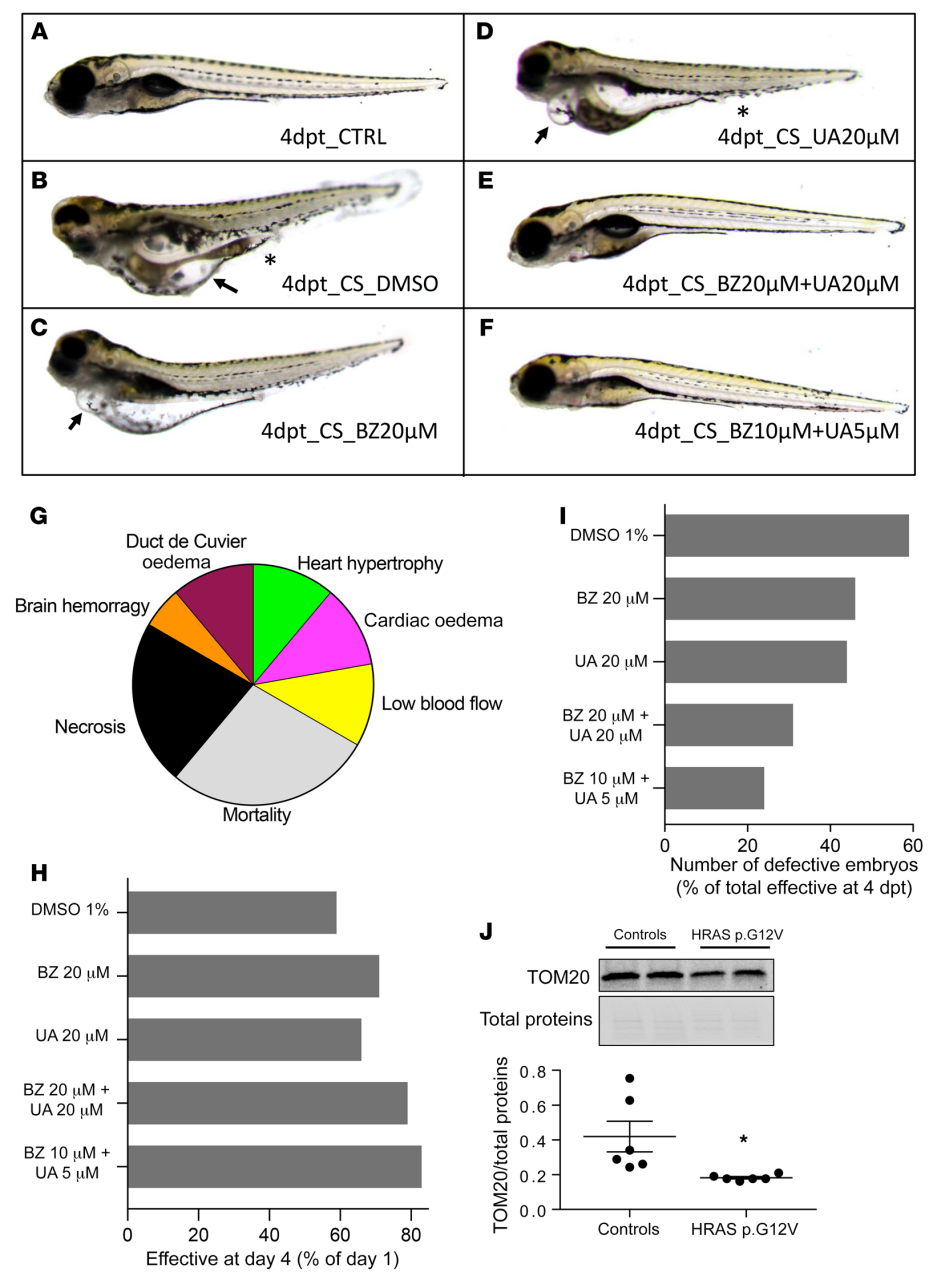
Combination of bezafibrate and urolithin A restores normal phenotype in Costello zebrafish. Phenotype analysis of (**A**) control zebrafish or (**B**–**H**) Costello zebrafish injected with HRASV12 plasmid at 5 dpf, after 4 days of treatment with (**B**) DMSO, (**C**) bezafibrate, (**D**) urolithin A, or (**E** and **F**) bezafibrate (BZ) and urolithin A (UA) in combination. Costello zebrafish developed edema (**B**–**D**, arrows) or hemorrhages and vascularization defects (**B** and **D**, asterisks). (**F**) Treatment with a combination of 10 μM BZ and 5 μM UA markedly reduced the percentage of abnormal embryos. (**G**) Illustration of the phenotypes observed in Costello zebrafish at 5 dpf after expression of HRASV12 plasmid. (**H**) Expression level of the mitochondrial protein TOM20 in control or HRAS p.G12V embryos. TOM20 protein content determined by Western blot was normalized to the total protein content. (**I**) Survival rate of Costello zebrafish after 4 days of treatment with DMSO, bezafibrate, or UA alone or in combination. Data were normalized to day 1 of treatment. (**J**) Percentage of defect appearance in Costello zebrafish after 4 days of treatment with DMSO, bezafibrate, or UA alone or in combination. Data are expressed as number of embryos or as the mean value of TOM20 expression. Unpaired t test was used to compare the 2 groups of zebrafish (WT or Costello). *P < 0.05.

CS is a developmental disorder, so we tested the preclinical strategy of mitochondrial stimulation at an early stage of the disease. To this aim, we generated a *GFP-HRASV12* zebrafish model of CS (Figure 8), as previously described (45). Embryos were observed daily from 2 days post fertilization (dpf) to 5 dpf in order to analyze the effect of HRASV12 overexpression on physiological development (Figure 8, A–G). At 2 dpf, 22% of the HRAS p.G12V embryos died, and this rate increased to reach 60% mortality at 5 dpf (Figure 8H). At 5 dpf, among embryos that were alive, 60% presented developmental defects similar to those observed in humans and mice (Figure 8, G and I) as previously described (45). We observed a cardiac phenotype characterized by heart hypertrophy (12%) and cardia and pericardia edema (12%) associated with poorly developed heart and reduced blood flow (12%). Additional phenotypes were observed, including brain hemorrhage (6%) and vascularization defect leading to edema in the duct of Cuvier or malformation of the aorta or the vein in the tail region around the urogenital opening (12%). The interindividual variability observed within the CS zebrafish population could be explained by the genetic position effect of the HRAS p.G12V transgene, as controlled by the regulatory environment of the genomic integration sites. In order to analyze the effect of mitochondrial bioenergetics and organelle proteostasis modulators on the potential rescue of the observed HRAS p.G12V embryo developmental phenotype, BZ and/or urolithin A (UA) were added to the zebrafish medium (Figure 8, C–F). Animal phenotyping revealed that the BZ and UA treatments increased the survival rate up to 70% and 65% (Figure 8H), respectively, and decreased the number of defective embryos among alive embryos (45% and 43%, respectively) as compared with the vehicle-treated (DMSO) HRAS p.G12V embryos (60% of defective embryos; Figure 8, F and I). The combination of 10 μM BZ and 5 μM UA increased substantially the treatment efficiency by improving embryo survival up to +30% (Figure 8H) and reduced to a large extent (3-fold) the number of animals presenting genetic developmental defects (Figure 8, F and G), as compared with vehicle-treated embryos. Measurement of HRASV12 plasmid expression by analysis of the level of GFP fluorescence emission confirmed the stable expression of the transgene during the experiments and the absence of effect of the different treatments on the HRASV12 plasmid expression (Supplemental Figure 14A). Finally, molecular investigation of the CS zebrafish model using Western blot performed on whole embryos revealed a 2-fold reduced expression of the mitochondrial marker TOM20 (Figure 8J). The combination treatment composed of 10 μM BZ and 5 μM UA restored the expression level of TOM20 in the whole embryo and corrected the defective phenotype (Figure 8, F and I, and Supplemental Figure 14B). A proteomic study performed on whole zebrafish animals treated with 10 μM BZ and 5 μM UA (Supplemental Figures 15 and 16) revealed a mode of action composed of at least 3 molecular mechanisms. (a) Bioenergetic stimulation, consisting of a strong stimulation of fatty acid oxidation systems (very-long-chain 3-oxoacyl-CoA reductase-A, carnitine O-palmitoyltransferase, electron transfer flavoprotein subunit β, etc.), suggested the occurrence of an “oxidative shift.” Accordingly, the LXR/RXR pathway that controls fatty acid metabolism was activated by the treatment (Supplemental Figure 16B). (b) Proteostasis modulation, consisting of stimulation of mitochondrial proteostasis machinery, included upregulation of Hsp70, CLPP, YME1-like 1b, COX7A2, ubiquinol-cytochrome *c* reductase complex assembly factor 1, and DNAJ (Hsp40 cochaperone) as well as selected proteasome components. The 2 proteases CLPP and YME1L1 have a particular interest in our study, as their genetic inhibition induces cardiomyopathy (23, 24). (c) Activation of the acute-phase response signaling pathway shows a systemic response aimed at the restoration of tissue homeostasis. In particular, the level of angiotensinogen, the sole precursor of all angiotensin peptides, was increased by a factor of log_2_fold = 4 by the treatment. Angiotensin I was also increased by log_2_fold = 2.55. Angiotensin I has vasodilator, antidiuretic, antithrombotic, and cardioprotective effects. Finally, the acute-phase response signaling stimulated the expression of members of the complement factors system, also involved in proteostasis. These findings suggest that the strategy of combined mitochondrial bioenergetics and proteostasis stimulation should be further investigated in CS.

## Discussion

Here, we performed a comprehensive analysis of the molecular determinants of CS using untargeted proteomics, bioenergetics, and molecular biology methods on a collection of biological models: tissues of a HRAS p.G12S CS mouse model, skin fibroblasts from patients with CS, CS hiPSC-CMs, human skin fibroblasts transduced with mutant forms of HRAS p.G12S and HRAS p.G12A, and a HRAS p.G12V zebrafish model of CS. Confrontation of these models allowed us to better understand the molecular impact of mutant HRAS in different contexts and to reveal tissue-specific changes. The original CS mouse model used in our study was generated by a knockin of a mutant HRAS p.G12S gene in C57BL/6 animals and developed HCM. All the CS mouse and zebrafish models reported in the literature develop HCM (7, 10, 16), and this clinical feature can be prognostic of worsening disease for patients.

Using in vitro and in vivo CS biological models, we discovered that germline or somatic HRAS p.G12S and p.G12A mutations alter mitochondrial proteome homeostasis and OXPHOS in a tissue-specific manner. For instance, CS mice exhibited downregulation of OXPHOS components in the heart and the liver, while the brain and, to a lesser extent, the skeletal muscle, showed accumulation of ETC proteins. The CS ectopic cell models (HRAS p.G12A and p.G12S) also exhibited a profile of OXPHOS protein accumulation associated with defective mitochondrial function. We further validated these findings in cardiomyocytes generated from the differentiation of hiPSCs from patients with CS. Taken together, our observations raised the hypothesis of defective mitochondrial homeostasis in CS.

Thus, we investigated the potential implication of AMPK in CS molecular pathophysiology and mitochondrial dysfunction, based on the pathway analysis performed on the differential proteome determined in the different CS mouse tissues and cellular models. Biochemical studies also showed that AMPK controls mitochondrial protein biogenesis and degradation (46, 47). AMPK dysfunction and mitochondrial respiratory chain defects are also frequent causes of HCM (17–22, 48). Our results revealed a strong inhibition of AMPK expression level (mRNA and protein) in heart samples from CS animals, in skin fibroblasts from patients with CS, in cell models overexpressing mutant forms of *HRAS*, and in CS hiPSC-CMs. These consistent findings support the hypothesis of defective AMPK signaling in CS. The interplay between RAS/MAPK and AMPK signaling was investigated in previous studies (49) and revealed the inhibition of the latter by the former, by at least 3 mechanisms: (a) LKB1 inhibition, (b) inhibition of AMPK catalytic activity, and (c) inhibition of AMPK by KSR2. However, little is known about the interplay between the HRAS/MAPK and the LKB1/AMPK pathway in the context of RASopathies. Our results revealed the inhibition of *PRKAA2* mRNA expression in skin fibroblasts from patients with CS, CS mouse hearts, and hiPSC-CMs. The α2 subunit of AMPK is essential for AMPK signaling because its phosphorylation on Thr172 activates the kinase. Moreover, a reduction of LKB1 protein content was also found in skin fibroblasts from patients with CS and CS mouse hearts, suggesting a dual inhibition of the LKB1/AMPK signaling pathway in CS. The observed reduction of LKB1_Ser428 phosphorylation level in fibroblasts from patients with CS and in CS mouse hearts suggests an even more upstream regulatory mechanism, whereby altered activity of kinases upstream of LKB1 might contribute to the decreased AMPK (thr172) phosphorylation observed. Moreover, LKB1 has numerous phosphorylation sites and the sole change in the phosphorylation level of Ser428 is not sufficient to evaluate LKB1 activity. Alterations in LKB1 interactions with other proteins or changes in its subcellular distribution could also occur.

We also discovered that the inhibition of *PRKAA2* mRNA expression by HRAS p.G12S was mediated by micro-RNA-211-5p (miR-221*). Thus, we searched for a possible effect of miR-221* on *PRKAA2* expression in the context of CS. We observed that inhibition of miR-221* rescued cellular energy homeostasis, suggesting that anti–miR-221 strategies could be considered in future studies. However, we were not able to detect the endogenous level of miR-221* in the hearts of WT or CS mice. Technically, the total pool of microRNAs was extracted in sufficient quantity and quality from the CS mouse heart, but amplification of miR-221* with a validated commercially available primer (Qiagen miScript Primer Assay) could not reach the detection threshold. One explanation for this finding could be that the microRNA miR-221-3p is differentially regulated in mouse and human cardiac pathology (50). Moreover, genetic studies investigating the role of miR-221 in the heart made use of transgenic mice overexpressing miR-221, not taking into account endogenous miRNA levels, as we tried to do (51–53). Finally, comparative analysis of microRNAs between humans and mice showed a significant level of divergence in the heart (54). For these reasons, we restrained our explanation of the molecular mechanism responsible for the mitochondrial defective phenotype of CS to the HRAS-mediated inhibition of LKB1/AMPK signaling. However, additional studies will be required to fully understand how altered LKB1 and AMPK signaling and miR-221* increased expression could be responsible for the broad spectrum of clinical symptoms of CS. miR-221* targets downregulated in fibroblasts from patients with CS could also play a role in CS pathophysiology, but this mechanism remains to be demonstrated in CS mouse heart. miR-221* targets are involved in cell migration, proliferation, and adhesion, key processes required for neural crest cell patterning in craniofacial development. Future studies could consider the role of miR-221* in the determination of the craniofacial features common in CS.

A major impact of the LKB1/AMPK inhibition observed in the different CS models investigated in our study is the alteration of mitochondrial proteostasis, albeit with tissue-specific differences. The causality link between AMPK signaling and the control of mitochondrial proteostasis was established in previous studies (46, 55, 56). Our analysis of the molecular mechanisms underlying the alteration of mitochondrial proteome and bioenergetics in skin fibroblasts from patients with CS and CS mouse hearts showed the inhibition of key executors and regulators of mitochondrial turnover to be AMPK, SQSTM1/P62, and CRIF1. These proteins play a role in mitophagy, and biochemical studies have shown that AMPKα2 coordinates this process and thereby protects against heart failure (57, 58), in complete agreement with our findings. Mechanistically, we observed a tissue-specific alteration in the machinery responsible for mitochondrial proteostasis, a very complex and intricate network of quality and quantity control mechanisms (59) recently considered as a therapeutic target in myocardial infarction (60). In the CS cell models, we further observed the altered expression of the autophagy regulators (ATG7) and targeted mitochondrial degradation (CLPX) but also that of central mediators of mitochondrial biogenesis (such as TFAM), suggesting defective mitochondrial proteostasis and biogenesis in cells from patients with CS. Our hypothesis was confirmed by the observation of reduced autophagic flux in cells from patients with CS, accumulation of mitochondrial proteins, and defective organelle bioenergetics. The mitochondrial protein CLPX reduced in cells from patients with CS is the ATP-dependent Clp protease ATP-binding subunit. The ClpP-ClpX (CLpXP) complex is a central regulator of mitochondrial proteostasis and bioenergetics (27, 28, 61, 62). Our observation of CLPX downregulation by mutant *HRAS* in CS could suggest further study of the link between the RAS/MAPK pathway and CLpXP.

The variable alteration of the mitochondrial proteostasis machinery and of the mitochondrial proteome itself, observed between the different tissues of the CS mouse, raised the question of the tissue-specific impact of *HRAS* germline mutations. There are still questions regarding the degree of functional tissue specificity exhibited by mutant *HRAS* in various cell types and tissues, and previous studies have shown that germline *HRAS* mutation alters the development of the different tissues to variable extents (63). For instance, the developmental phenotypes of patients with CS and CS animal models indicate a strong tissue-specific effect of the germline *HRAS* mutations on the craniofacial development, heart structure and function, skin, and predisposition to specific cancers. This tissue-specific impact of *HRAS* germline mutations was partly explained by the specific role of the different RAS isoforms in tissues of variable embryological origins (64, 65). Differences in the interactions with regulators and effectors of the RAS signaling pathway also participate to the tissue-specific functions of the different RAS isoforms. The LKB1/AMPK signaling involved in CS molecular pathophysiology is also a tissue-specific pathway (58, 66, 67). In particular, the control of mitochondrial biogenesis and degradation by AMPK is variable between tissues. We measured a reduced TFAM content in *HRAS* mutant cell models and in the CS mouse heart. However, the reduction in PGC1α was only detected in the mutant *HRAS* cell models. This result could be explained by the fact that PGC1α is typically regulated by deacetylation in muscle, rather than through changes in expression (68). Another tissue-specific concern of our study was the use of skin fibroblasts that present with very different bioenergetic features as compared with cardiomyocytes. Previous studies in the field of mitochondrial diseases have shown that skin fibroblasts do not recapitulate the bioenergetic dysfunction found in the skeletal muscle (69). The use of hiPSC-CMs and of CS mouse hearts was instrumental to validating our findings. In particular, we found that most of the ATP produced in CS hiPSC-CMs was obtained from glycolysis, as previously observed in conditions of cardiac disease and OXPHOS dysfunction (70, 71). However, in the context of CS, the skin fibroblasts constitute a relevant cell model, as the skin expresses the disease in various forms (72). Conversely, while hiPSC-CMs from patients with CS provide a pertinent model allowing comparison with CS mouse hearts and human hearts, the growth conditions of the cardiomyocytes will directly impact their bioenergetics and may not provide the best environment to study the mechanism underlying OXPHOS alteration. In particular, the medium used for the maintenance of hiPSC-CMs contains a large number of growth factors and high concentrations of energy substrates, which limits the study of the LKB1/AMPK pathway. To conclude, we think that studying the mechanisms in the CS mouse heart may provide a more reliable data set for understanding the pathophysiology of CS cardiac disease; the cell models used in our study, which all revealed a constitutive mitochondrial dysfunction, present specific bias for the mechanistic investigations. Moreover, our discovery of altered LKB1/AMPK signaling and defective mitochondrial proteostasis and bioenergetics only explains one facet of the complexity of CS molecular pathophysiology. For instance, *Prkaa2*-KO mice show no cardiac alteration, and these mice show exacerbated pressure overload–induced left ventricular hypertrophy only under stress conditions (73). Likewise, we found a small activation of AMPKα2 (p-thr172) and a consecutive low inhibition of ACC (p-ser79) in the CS mouse and cell models investigated under routine conditions.

In this study, we discovered a reduction of LKB1 protein content in CS mouse hearts and skin fibroblasts from patients with CS; this is in agreement with results from a previously published work that showed that genetic deletion of *LKB1* in the heart triggers hypertrophy and cardiac dysfunction (74). These findings suggest that AMPK inhibition alone cannot induce heart dysfunction and that additional stress, as mediated by the deregulated HRAS/MAPK pathway, or a challenging bioenergetic environment must occur. A series of transcriptomic targets have been identified in response to AMPK activation. For instance, AMPK was shown to phosphorylate the transcription factor FoxO3 on 6 serine residues to enhance its transcriptional activity (75) toward its target genes. Growing evidence suggests that in the long-term AMPK promotes metabolic reprogramming via effects on gene expression at least partly through regulation of specific transcription factors and transcriptional coactivators (31). In agreement with this view, analysis of CS heart proteome revealed a complex molecular signature of cardiac dilation and damage, apparently independent of AMPK signaling. Finally, previous metabolic investigation in a *HRAS p.G12S* CS mouse model (10) revealed a decrease in fatty acid oxidation enzyme expression in the liver, in agreement with the inhibition of LKB1/AMPK signaling observed in our study.

Finally, our work demonstrated that BZ could rescue or prevent the defective mitochondrial function in skin fibroblasts from patients with CS and CS mouse hearts. We discovered that the mode of action of BZ in vitro is multiple and involves the stimulation of PGC1α expression, mTOR inhibition, and activation of LC3II-mediated autophagy. In particular, BZ rescued the level of the mitochondrial proteostasis regulator ClpX, which was downregulated in CS mouse hearts and cell models. We treated the CS mouse with 0.05% BZ in the diet based on a study showing cardiac beneficial effects in a mouse model of the mitochondrial disease Barth syndrome (42). Other rodent studies using a higher dose of BZ also showed OXPHOS improvement and beneficial effects in various mouse models with mitochondrial dysfunction (40, 76–81). However, hepatomegaly was observed when using suprapharmacological doses of BZ in mice, although this effect was not observed in humans (40). BZ is well tolerated in humans, and clinical trials have revealed that this drug can reduce the heart rate and blood pressure in patients with hypertriglyceridemia (82). BZ also improved the condition of patients with hyperlipidemia (83) and fatty oxidation defect (41). Furthermore, a recent trial on 6 patients with a genetic mitochondrial disease revealed an improved cardiac function (84). To further evaluate the potential of mitochondrial bioenergetics and proteostasis modulation in the treatment of CS in the early stage of the disease we tested the efficacy UA alone or in combination with BZ in a previously described CS zebrafish model (45). The mitochondrial proteostasis modulator UA was recently characterized and showed no toxicity in a human clinical trial (85–87). We discovered that UA in combination with BZ synergistically increased the survival of CS embryos and significantly reduced the occurrence of developmental defects. The mode of action of this combination of drugs involved the stimulation of fatty acid oxidation, the activation of several mitochondrial proteostasis regulators and executors, and the stimulation of the acute-phase response, which involved regulation of the angiotensin system. Of interest for our study, previous work showed that AMPK regulates the renin-angiotensin system, and the observed alteration of AMPK signaling in CS could also participate in the cardiac disease through this system (88).

Therefore, our study provides preclinical and molecular evidence that stimulation of mitochondrial quantity control and bioenergetics, as obtained with BZ and UA treatment, improved the health of cellular and animal models of CS. A more detailed heart structure and function analysis with additional clinically relevant data will be required before the use of BZ and UA in combination for the prevention of cardiac disease in CS is proposed. To conclude, our work supports the view that mitochondrial quality surveillance is a therapeutic target in heart dysfunction in CS.

## Methods

Further information can be found in Supplemental Methods.

### Study approval.

All patients gave written informed consent to participate in this study, according to the Committee for the Protection of Persons (University of Bordeaux, Bordeaux, France) (IDRCB no. 2015-A00705-44, CPP 2014/44, ClinicalTrials.gov NCT02812511). All the zebrafish studies conducted for this manuscript have been reviewed and approved by the Comité d’éthique Langedoc Roussilon (Université de Montpellier, Montpellier, France) (F341725/21063-2019061317218694). The mouse preclinical studies were performed in accordance with the European guidelines for animal experimentation at the Institut Clinique de la Souris, Illkirch-Graffenstaden, France. All the mouse studies conducted for this manuscript have been reviewed and approved by the comité d’éthique Com’Eth, Illkirch, France.

## Author contributions

RR, CH, TS, and LD participated in the conception and design of the study. LD and CH (gene and miR expression studies); LD, NDA, and NB (patients with CS and mutant HRAS bioenergetics properties); ED (respiratory chain enzymes activities); GBA, YH, and TS (BZ preclinical study on the CS mouse model); and CA and WB (proteomics) performed the experiments. RR and LD performed bioinformatics (IPA, Qiagen) and statistical (Prism) analyses of the data. VGP produced the lentiviral plasmid for HRAS mutant ectopic expression. PE participated in the bioenergetics analyses. SC and MB provided expertise in proteomics. PES and JBT performed the cardiology investigations on patients with CS. LF and KK performed the zebrafish study at Azelead. FM, NL, MD, and LB produced the hiPSCs and their differentiation in cardiomyocytes. They also performed Western blotting on these cells. DL performed diagnostics studies, genetic identification, and patient management. DL, NB, and RR obtained the agreement to generate the cell lines from patients with CS used in this study (ClinicalTrials.gov NCT02812511). RR supervised the entire project and wrote the paper along with LD and NDA, with suggestions from all authors.

## Supplementary Material

Supplemental data

## Version 1 03/01/2022

In-Press Preview

## Version 2 04/15/2022

Electronic publication
