# Supplementary material for: HRAS germline mutations impair LKB1/AMPK signaling and mitochondrial homeostasis in Costello syndrome models
Source: J Clin Invest. 2022 Apr 15;132(8):e131053. doi: 10.1172/JCI131053 (PMC9012293; doi:10.1172/JCI131053)
Supplement: Supplemental data [file jci-132-131053-s133.pdf]

Costello patients		P1	P2	P3	P4	P5	P6	P7	P8	P9	P10	P11	P12	P13	P14
**Age**		13	29	7	7	23	10	6	25	21	22	17		29	20
**HRAS mutation**				G12A		G12S	G12A		c.187_207dup			G12S		G12S	
**Age at diagnosis (month)**		1			8	10	6		14	4.5		14		17	
**Sex**		F	F	M	M	M	F	F	M	F	F	M	M	F	F
**Growth and development**															
	Birth weight > 90 centile	-			-	+	-		-	-				+	
	Feeding problems in infancy	+		+	+	+	+	+	-	-			+	+	
	Relative macrocephaly				+	-	-		-	-			+	+	
	Short stature				+	+	+		+	-				+	
	Psychomotor retardation	+			+	+	+		-	+					
**Dysmorphic features**															
	Curly/ sparse hair				+	+	+	+	+		+	+	+	+	+
	Coarse facial features				+	+	+	+	+		+	+	+	+	+
	Warts/ papillomas (facial)				-	-	+	-	+	+	-	+	-	+	-
	Hyperkeratosis				+	-	+	-	+		+	+	-	+	
	Low-set ears				+	+	+	+	+		+	+	+	+	+
	Rendundant / loose skin				+	+	+	+	-		+	+	+	+	
	Low posterior / trident hairline				-	-	+	+	-		+	+	+	+	+
	Dystrophic nails				-	-	+	-	+		-	-	-	+	
**Organ dysfunction**															
	Hoarse voice														
	Cardiomyopathy	+	+	+	+	+	+		+	+				+	
	Tonic-clonic seizures				-	-	-		-	-				-	

Table S1: Costello patients enrolled in the 'COSMIT' study (ClinicalTrials.gov Identifier: NCT02812511)

Cardiac investigation in Costello patients		**n (%)**
	patients	**10**
	Age (years)	**11.9 ± 8.1**
	Sex ratio	**1/1**
Transthoracic echocardiography		
	Normal	**3 (30)**
	Pulmonary stenosis	**0 (0)**
	Mitral insufficiency	**0 (0)**
	Left ventricular hypertrophy	**7 (70)**
	Left ventricular obstruction	**2 (20)**
	Atrial septal defect	**0 (0)**
Electrocardiogram		
	Normal	**4 (40)**
	Right atrial hypertrophy	**2 (20)**
	Right ventricular hypertrophy	**0 (0)**
Age is expressed as the mean ± standard deviation.	Left ventricular hypertrophy	**0 (0)**
	Q wave (pathologic)	**4 (40)**
	Abnormal axis	**5 (50)**

Table S2: Cardiac parameters of the Costello patients (COSMIT) study (ClinicalTrials.gov Identifier: NCT02812511)

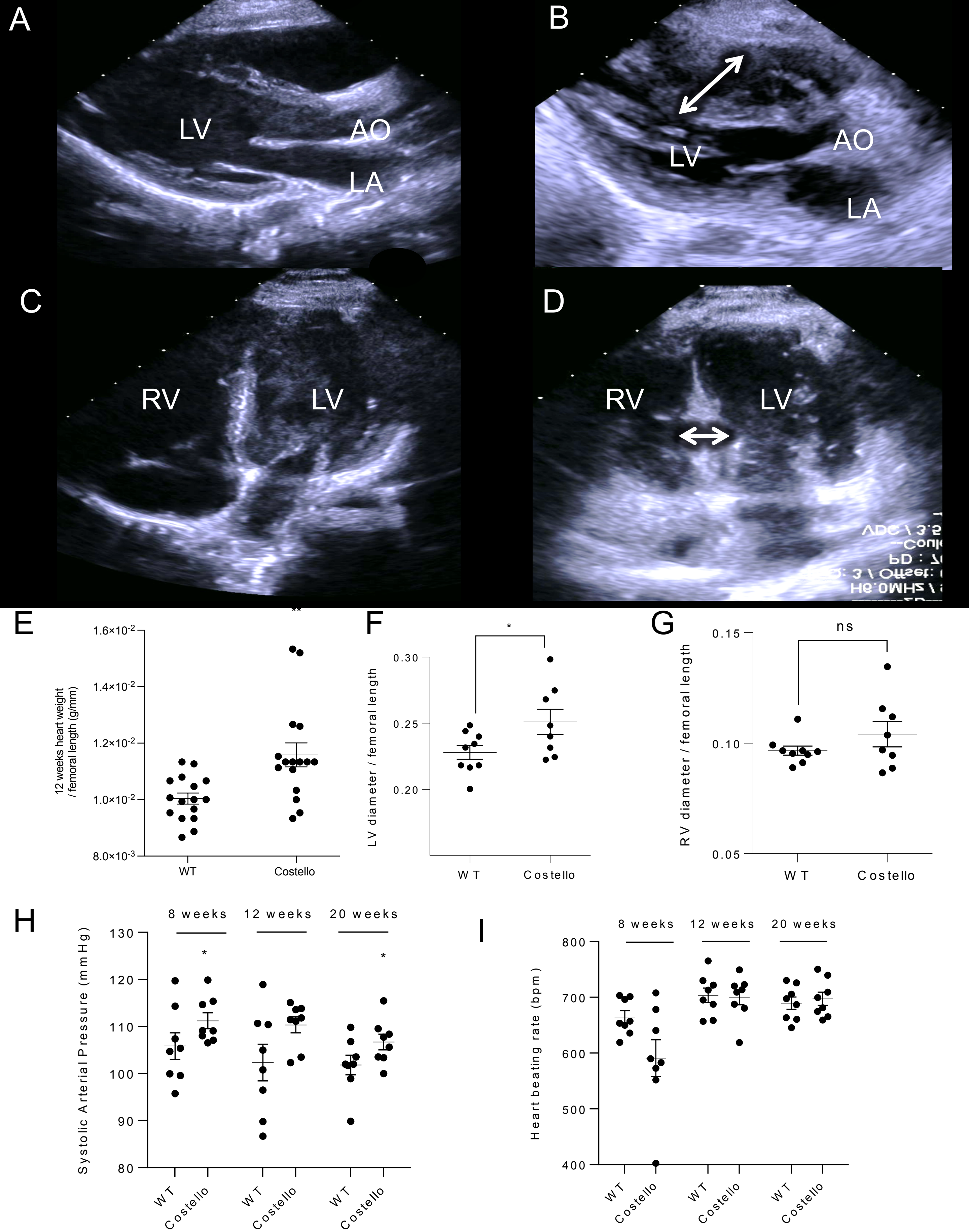

Figure S1: Hypertrophic cardiomyopathy (HCM) in CS patients and mouse. Parasternal (**A,B**) or apical '4 cavities' (**C,D**) echography was performed in controls (**A,C**) or Costello patients (**B,D**). Left ventricle hypertrophy was shown by the arrows. (**E**) Heart mass measured in twelve-weeks old animals was normalized to femur length (n=16). (F) Left ventricle diameter normalized to femur length was measured in twelve-weeks old animals (n=6). (**G**) Right ventricle diameter normalized to femur length was measured in twelve-weeks old animals (n=6). Systolic blood pressure (**H**) and heart beat rate (**I**) was measured at 8, 12 and 20 weeks of age (n=8). The data were expressed as the mean ± SEM, *p < 0.05, **p < 0.01 (unpaired t-test).

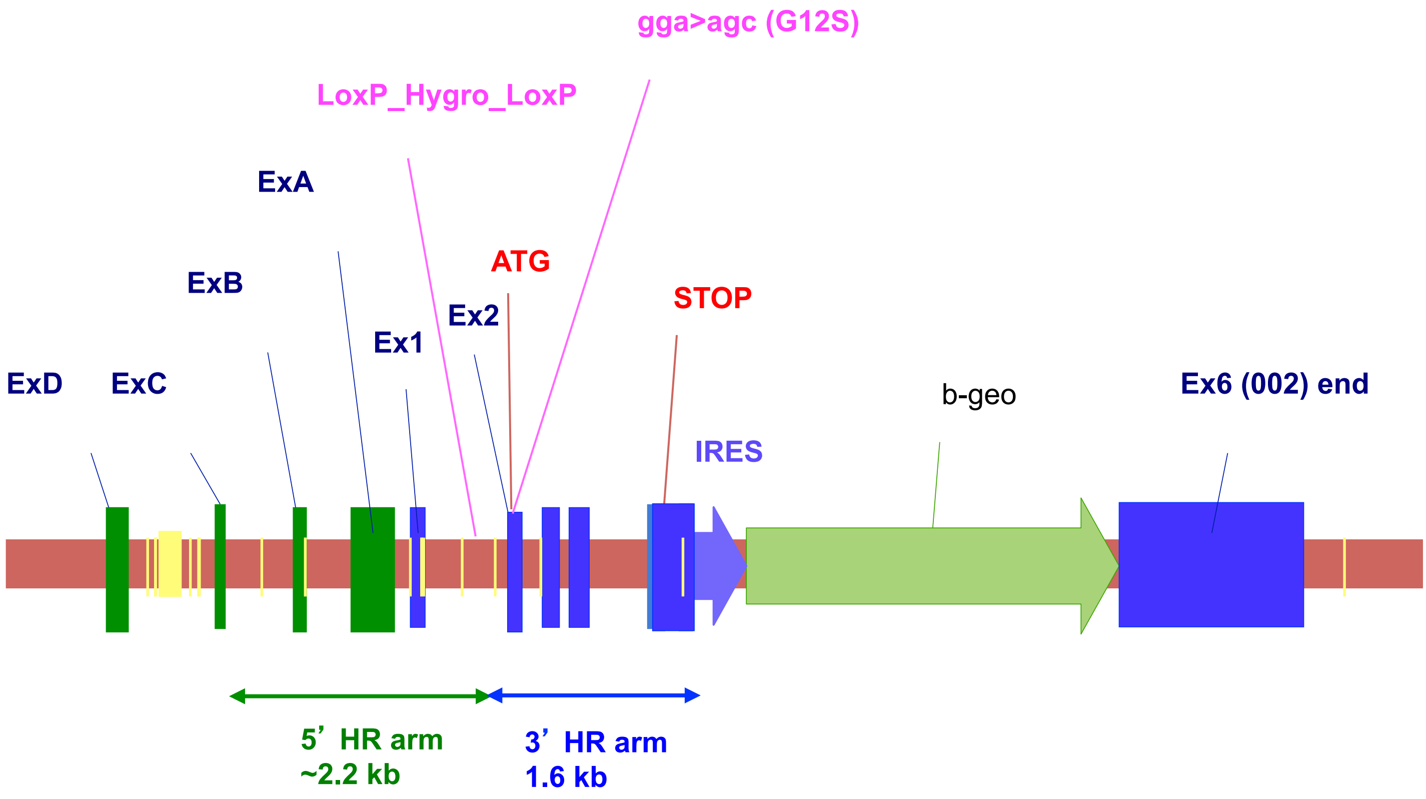

Figure S2: HRAS p.G12S mouse model. Mutated locus of the *Hras* gene. Gene construct used for the generation of the HRAS p.G12S ‘Costello’ mouse model. (MCI code : K479/ IR 1850).

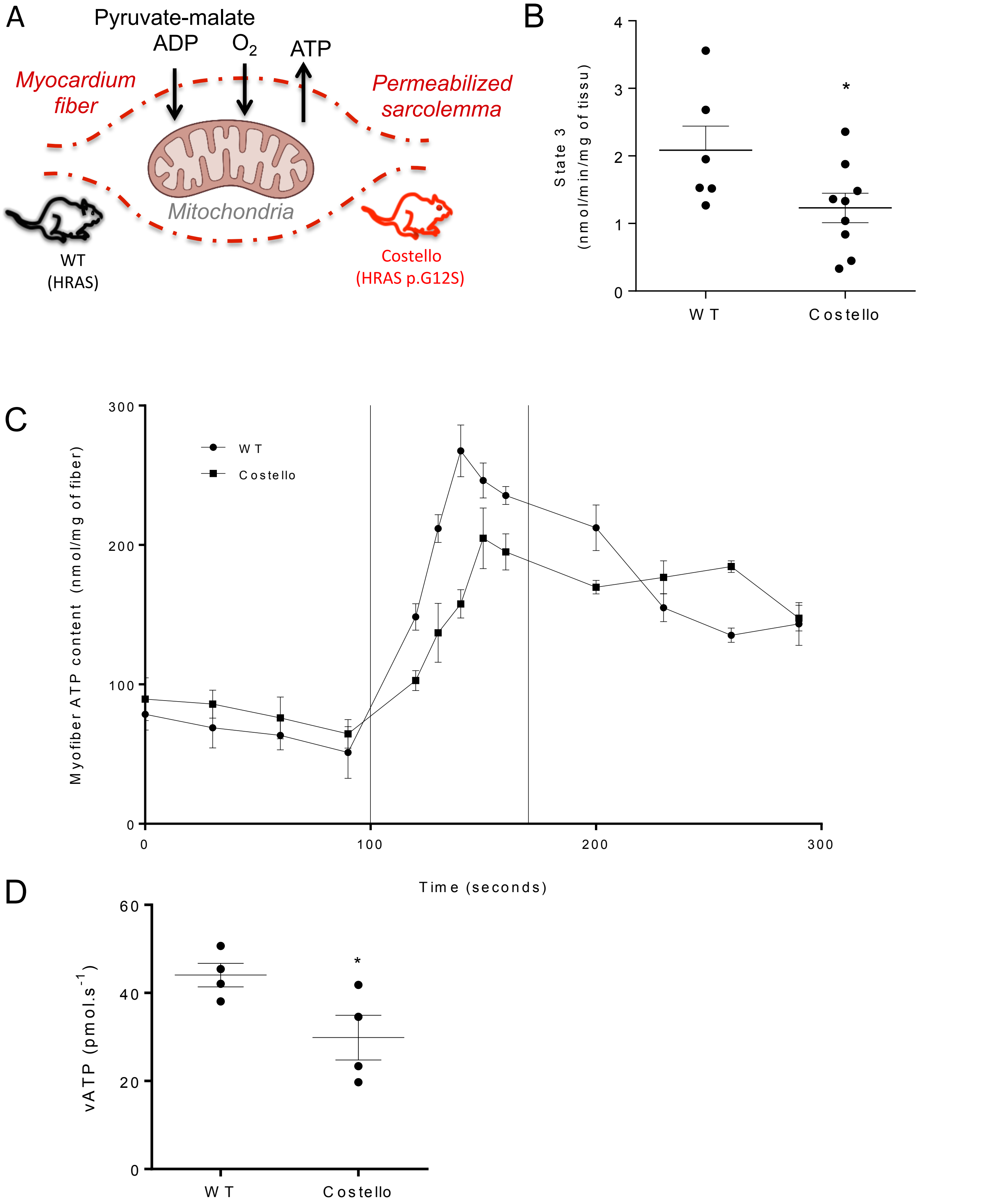

Figure S3: Alteration of skeletal muscle mitochondrial bioenergetics *in situ* in the HRAS p.G12S Costello mouse model. (**A**) Permeabilized skeletal muscle fibers methods allowing the control of energy substrates used for mitochondrial respiration determined *in situ* (**B**) State 3 respiration measured in presence of pyruvate-malate and ADP. (**C,D**) ATP synthesis rate measured on permeabilized muscle fibers. After addition of ADP (at 100 seconds) the ATP produced was quantified by bioluminescence every 30 seconds. Then, OXPHOS was inhibited with Antimycin A + Oligomycin + rotenone, and the rate of ATP hydrolysis was measured. The data are expressed as the mean ± SEM (N=3) , *p < 0.05 (unpaired t-test)

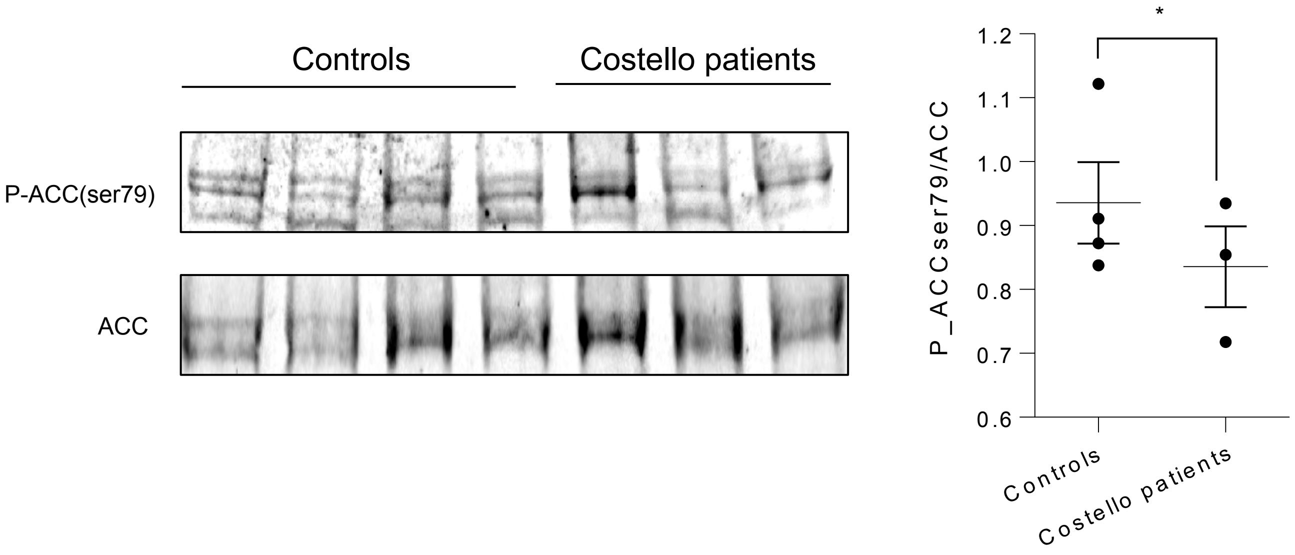

Figure S4: Alteration of ACC phosphorylation in human skin fibroblasts obtained from Costello patients. The expression levels of ACC and P-ACC(ser79) were determined by westernblot and the P-ACC/ total ACC ratio was calculated (mean value ± SEM). A unpaired t-test was performed to determine the p-value (* $p<0.05$).

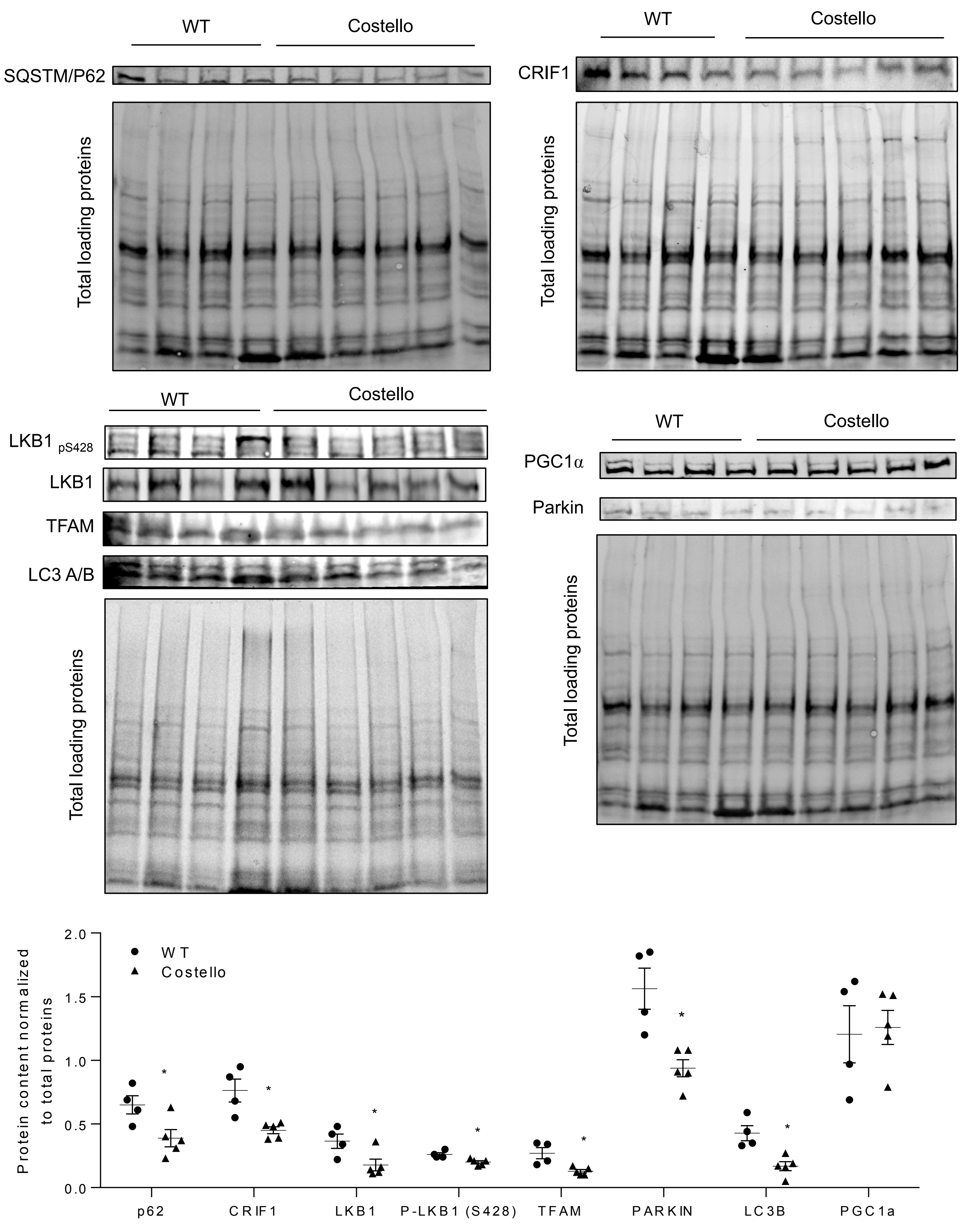

Figure S5: Altered expression of mitochondrial proteostasis executors in the heart of Costello mice at 10 weeks of age. The expression level of HSP70, P62, LKB1, P-LKB1(S428), TFAM, LC3(A/B), CRIF1, and Parkin was determined using westernblot in the heart from Costello mice (HRAS p.G12S) or wild-type (WT) animals. The expression level of each protein was normalized to the total protein content loaded in each lane. The data were expressed as the mean value ± SEM. *p<0.05 (unpaired student t test).

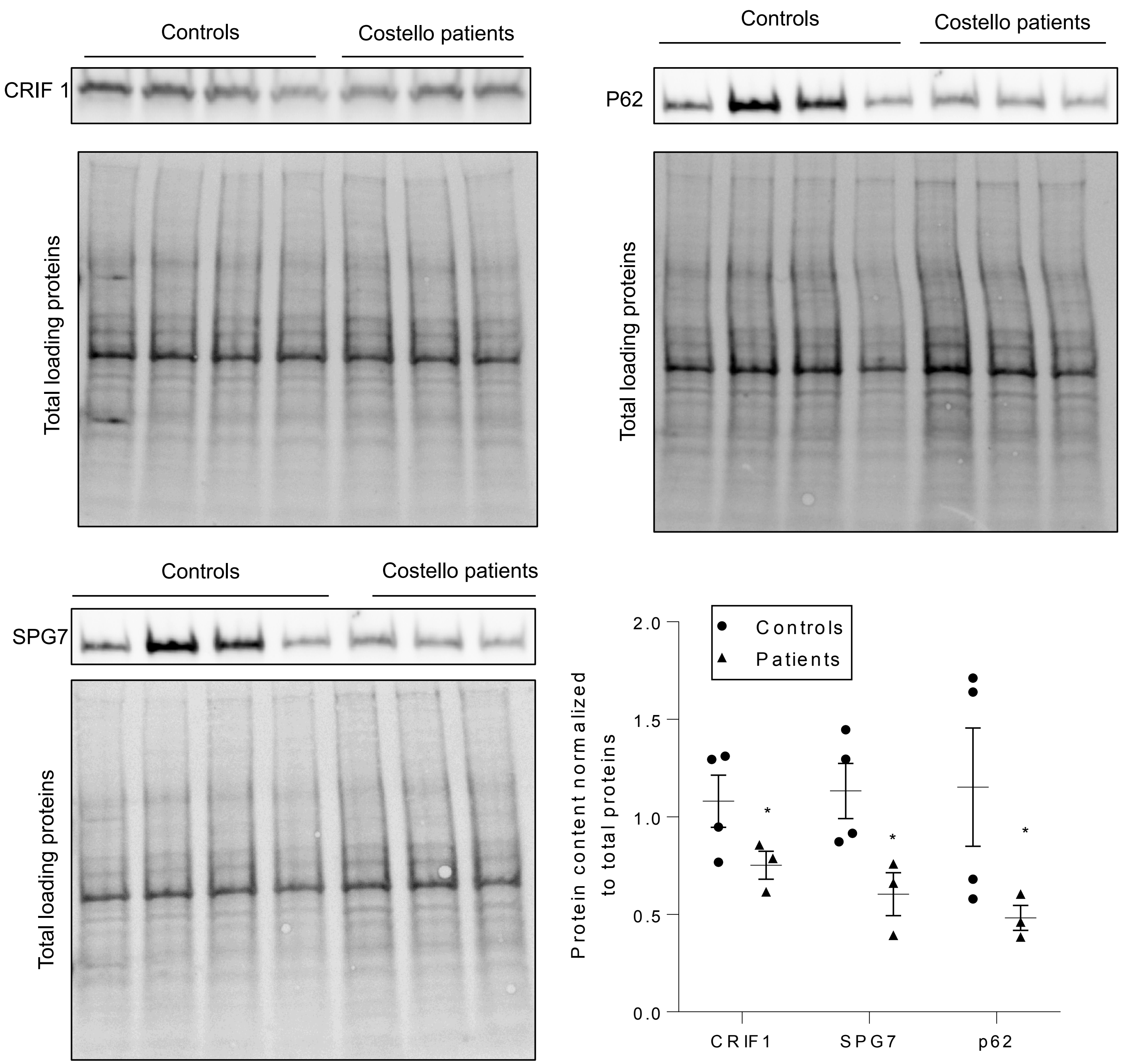

Figure S6: Altered expression of mitochondrial proteostasis executors in human skin fibroblasts obtained from Costello patients or control individuals. The expression level of CRIF1, P62 and SPG7 was determined using westernblot in controls or Costello patients. The expression level of each protein was normalized to the total protein content loaded in each lane. The data were expressed as the mean value ± SEM. *p<0.05 (unpaired student t test).

A

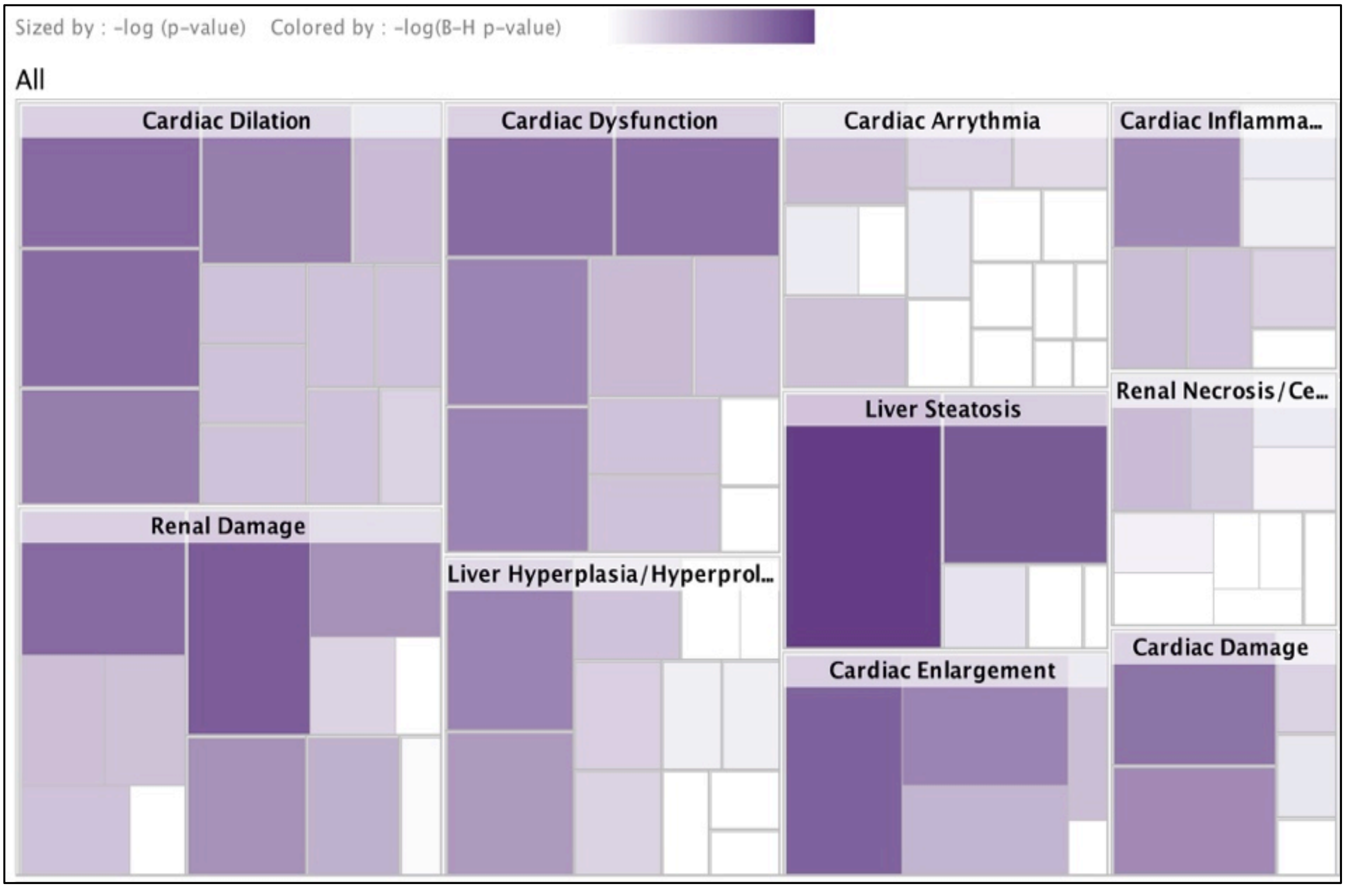

B

Heart Disease Category	p-value	Molecules
Hypertrophy of heart	1,70E-06	CKM,CKMT2,CPT2,CRYAB,CSRP3,FN1,MB,MYBPC3,POSTN,PPIA,SLC25A4,SOD2,TLN1,TTN
Fibrosis of heart	2,23E-06	ACSL1,APOA1,CSRP3,FN1,IGHM,LMNA,MYBPC3,POSTN,SOD2,TLN1
Dominant dilated cardiomyopathy	4,90E-06	CRYAB,CSRP3,LMNA,MYBPC3,TTN
Primary dilated cardiomyopthy	6,85E-06	ACADVL,CRYAB,CSRP3,LMNA,MYBPC3,TTN
Left ventricular dysfunction	8,89E-06	APOA1,CKM,CKMT2,CPT2,LMNA,MB,MYBPC3,TTN
Dysfunction of heart	1,01E-05	APOA1,CKM,CKMT2,CPT2,CRYAB,LMNA,MB,MYBPC3,TTN
Cardiotoxicity	1,76E-05	ANXA1,CAT,CBR1,RAC2,TUBA1A
Dilated cardiomyopathy	4,31E-05	ACADVL,ACSL1,CRYAB,CSRP3,FN1,LMNA,MYBPC3,SOD2,TLN1,TTN
Dilated cardiomyopathy 1A	4,33E-05	LMNA,MYBPC3
Hypertrophy of left ventricle	6,97E-05	CKM,CKMT2,CPT2,MB,MYBPC3,TTN
Left ventricular noncompaction	7,67E-05	LMNA,MYBPC3,TTN
Carditis	1,30E-04	ANXA1,CKM,IGHM,MYBPC3,TUBA1A
Damage of heart	1,48E-04	ANXA1,CBR1,HADHA,MYBPC3,POSTN
Interstitial fibrosis of heart	8,17E-04	ACSL1,CSRP3,POSTN
Myocardial infarction	8,46E-04	APOA1,C3,CA3,FGB,FGG,MB,POSTN,TUBA1A

Figure S7: Proteins altered in the Costello mice heart and involved in cardiac diseases. (**A**) the search for proteins involved in various aspects of cardiac disease was performed on the differential proteome dataset obtained on Costello mice heart, using the ‘disease’ module of Ingenuity Pathway analysis (Qiagen). (**B**) List of proteins involved in cardiac dilation and cardiac dysfunction altered in expression in the Costello mouse heart. The p-value of the different heart disease categories enriched in the Costello mouse heart proteome is shown, as well as the list of corresponding proteins.

A

Mitochondrial Proteome Maintenance Mechanism	***Main actors (proteins in yeast or (mammals)).***
Mitochondrial protein translocation associated degradation (**mito-TAD**)	Cdc48 (P97/VCP); Ubx2 (UBXD8) ;
Mitochondria compromised import response (**mitoCPR**)	Cis 1 ; Msp1 (ATAD1) ; TOM20
Mitochondria localized associated-ribosome quality control (**MitoRQC**)	Vms1 (ANKZF1), Ltn1, RQC2, hul5
Mitochondria unfolded protein response (**mitoUPR**)	ATFS-1 (ATF5) ; LON, Hsp60 ; ClpP ; ATF4, CHOP, OMA1, Dele1,
Mitophagy	Pink1, PArkin, Tim23, PArl, USP30, PHB2, LC3, Fundc1, p62, HSP70
Proteolysis of mitochondrial proteins	YME1L1 and its targets [PRELID1, PRELID3b, STARD7], OPA1, SPG7-AFG3L2, OMA1, LONP, CLPP, CLPX, PREP, ERAL1

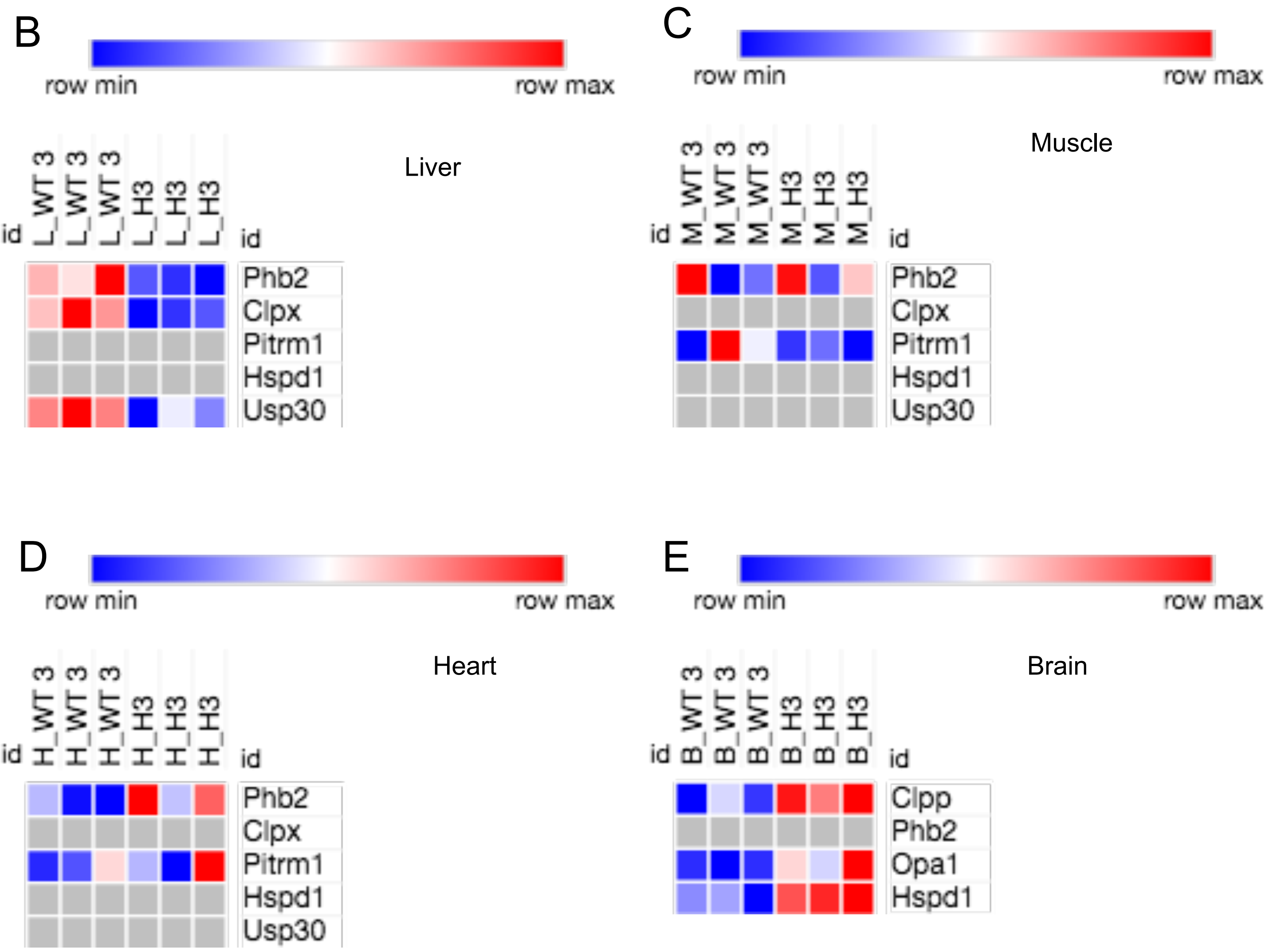

Figure S8: Tissue-specific alteration of mitochondrial proteostasis in the Costello mice. The expression level of a series of proteins involved in a very complex and intricated network of quality and quantity control mechanisms of mitochondrial proteins (**A**) was determined by mass spectrometry in the liver (**B**), skeletal muscle (**C**), heart (**D**) and brain (**E**). For each tissue, 3 wild-type animals (WT3) were compared to three heterozygous HRAS p.G12S mice (H3), all taken at 3 weeks of age.

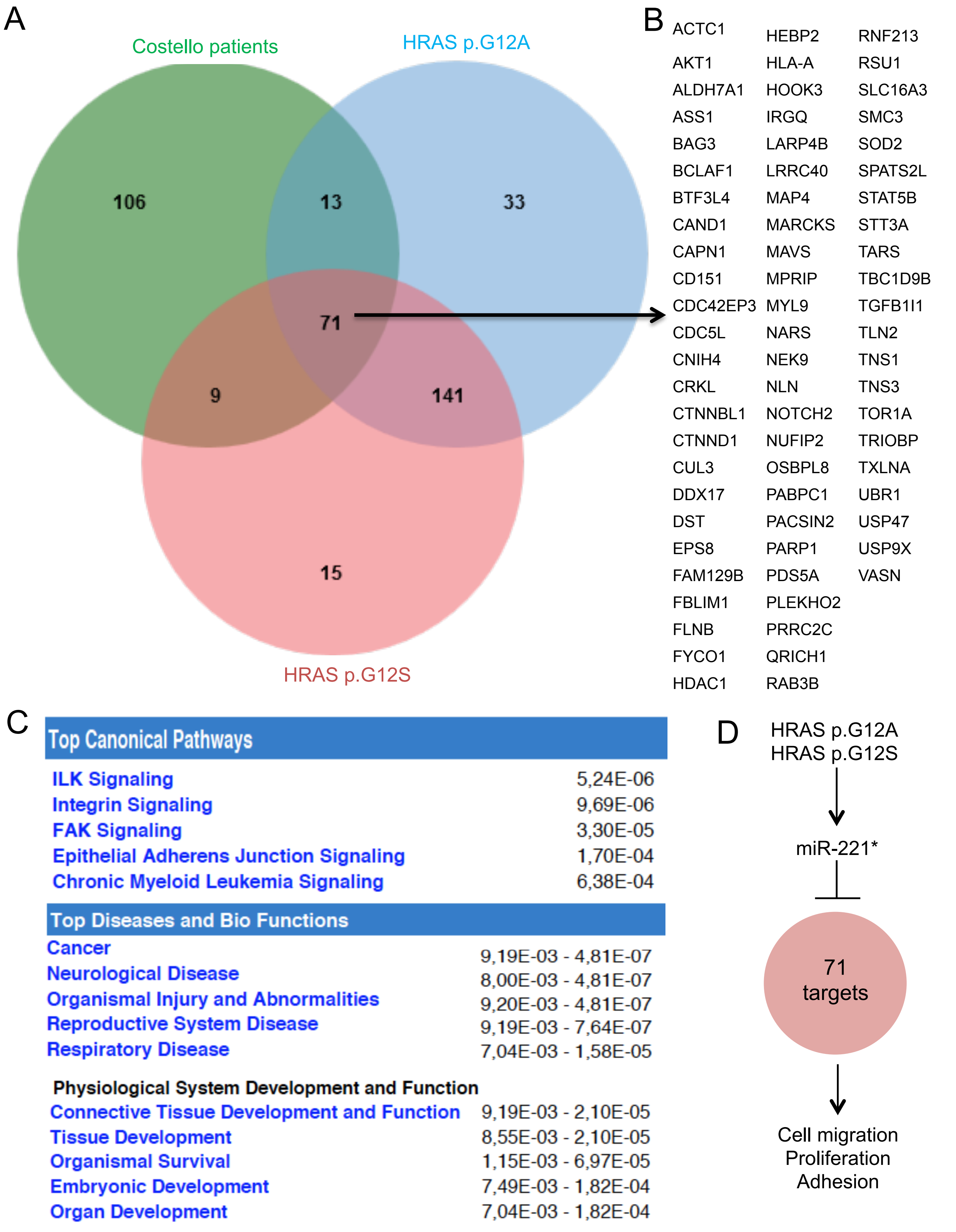

Figure S9: **miR-221-5p targets downregulated in skin fibroblasts from Costello patients and in the related cell models. (A)** Venn diagram of the protein targets of miR-221* downregulated in the proteome of Costello patients' cells and mutant HRAS p.G12A and HRAS p.G12S expressing fibroblasts. (**B**) List of the common targets of the miR-221* downregulated in the CS patient's fibroblasts and CS cell models. (**C**) Pathway analysis of the 71 proteins targets of the miR-221* and downregulated in the CS models. (**D**) Schematic summary of the cellular functions altered by the targets of the miR-221* downregulated in the CS cells models (n=3).

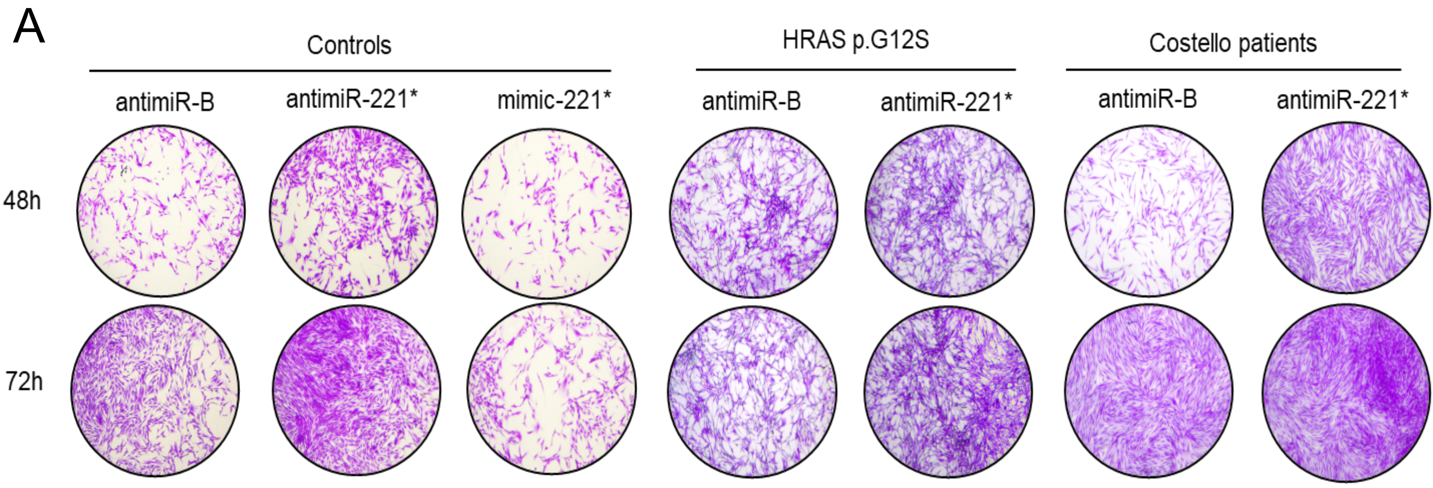

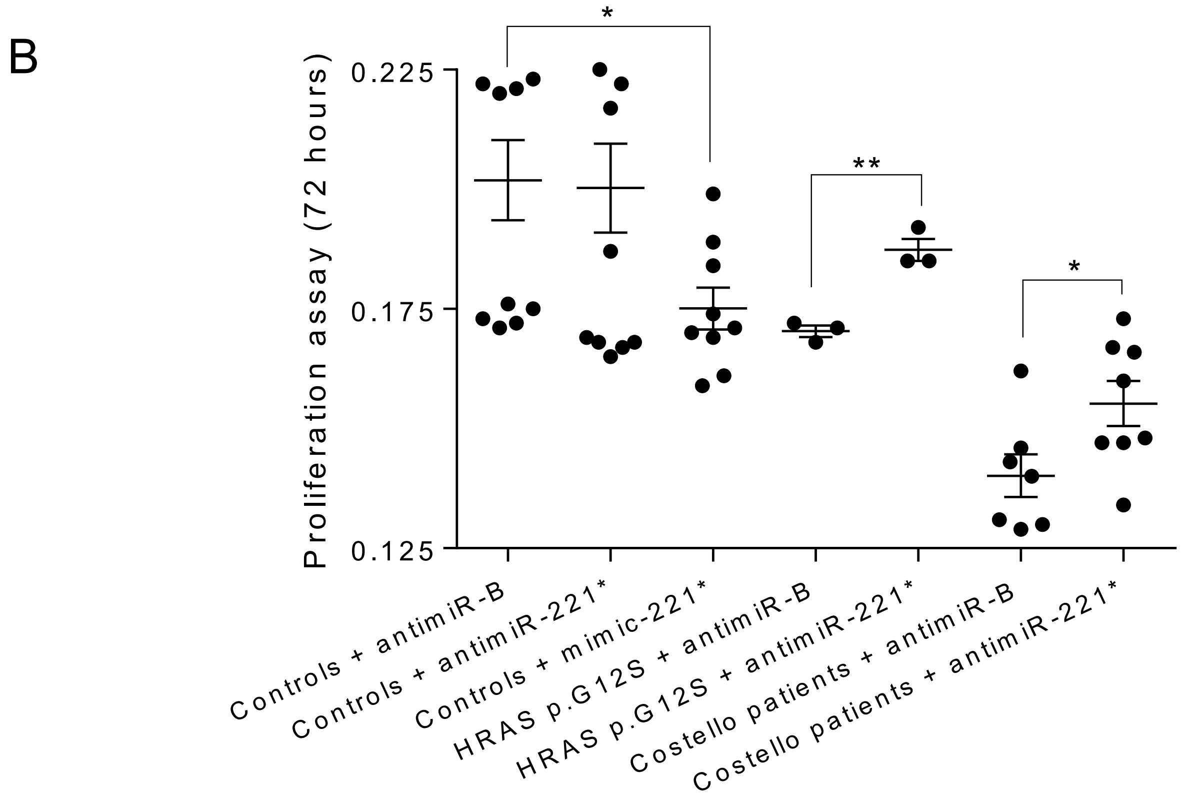

Figure S10: **miR-221* impairs cell survival in oxidative growth medium.** Effect of the anti-miR-221* and the mimic-221* on human skin fibroblasts cell growth in oxidative medium (glucose-deprived medium). (**A**) Representative images obtained using the SRB cell enumeration assay. (**B**) Quantification of the results. The data (n=3) were expressed as the mean ± SEM. One way-Anova with Dunnett's correction was used to compare the effect of the anti-miR-221* with the control miR (antimiR-B) in the different types of cells.
*p < 0.05, **p < 0.01.

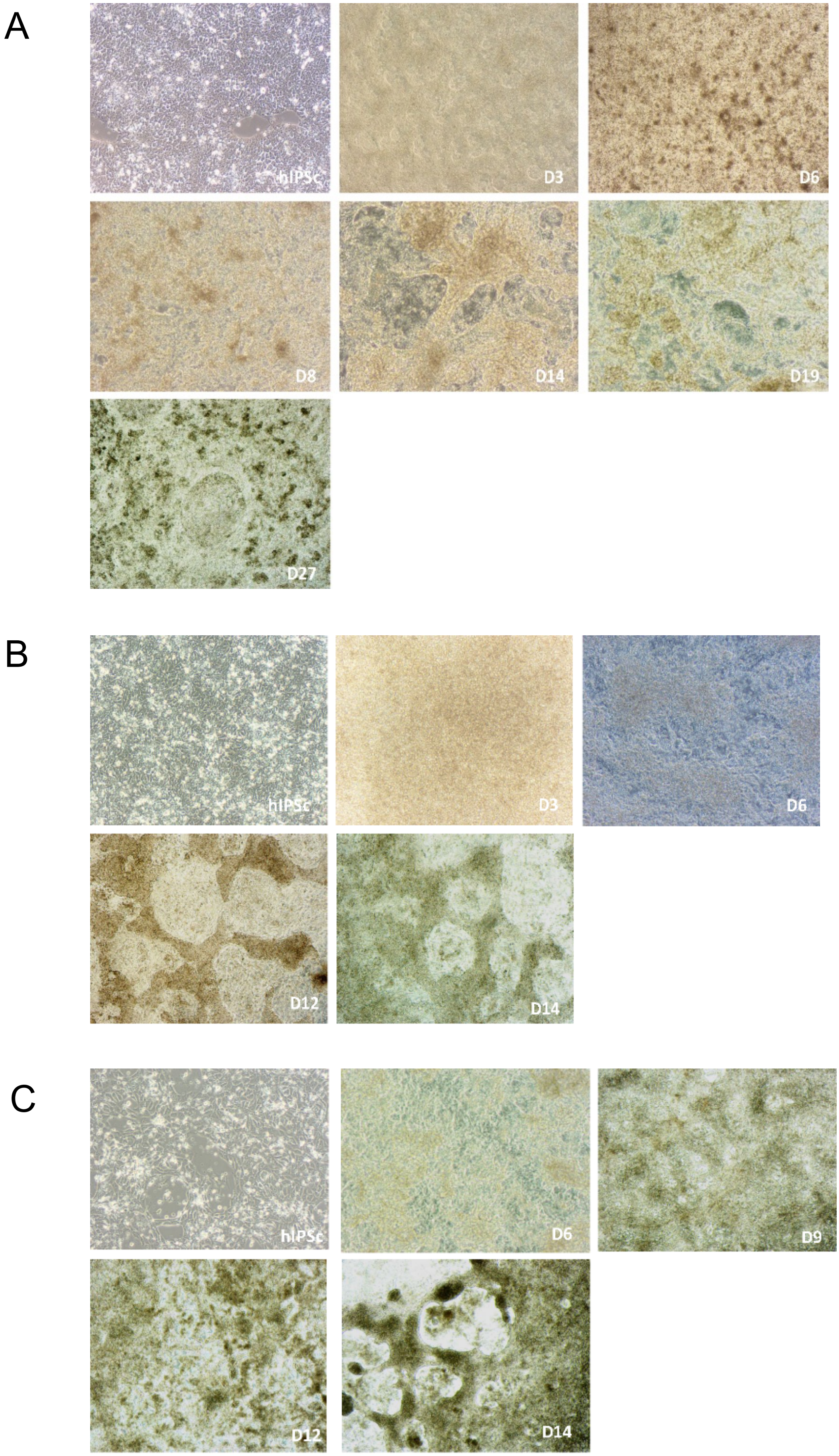

Figure S11: **Histology staining of the CS-hiPSC derived human cardiomyocytes.** (**A**) Control cell line hIPSC_CM AG08H (first cardiomyocytes beating were recorded at D08). (**B**) Control cell line hIPSC_CM AG161B (first cardiomyocytes beating were recorded at D12). (**C**) Costello cell line CM_Cos HRAS p.G12A (first cardiomyocytes beating were recorded at D9).

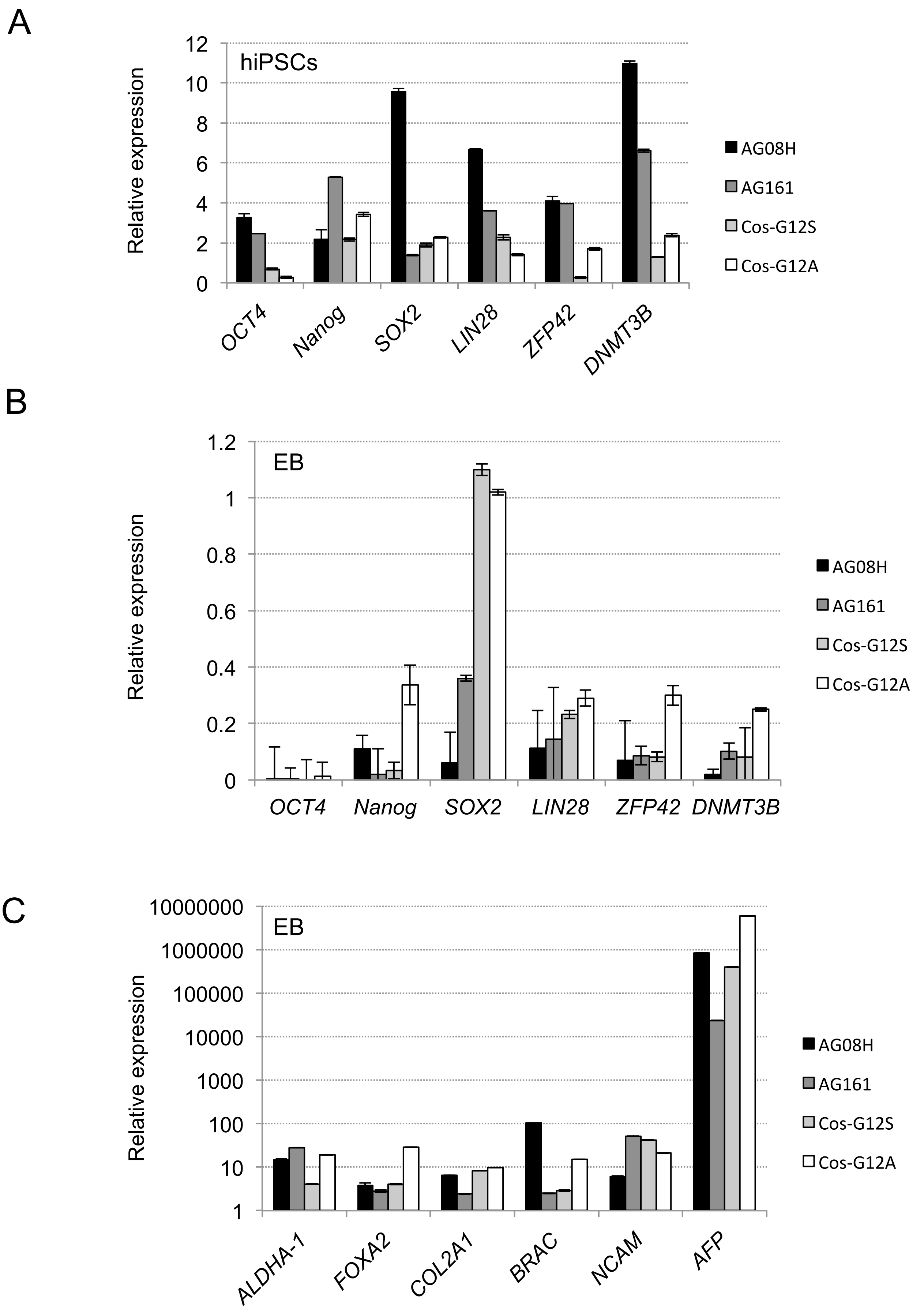

Figure S12: **Generation of the CS-hiPSC and derived human cardiomyocytes.** The control hIPSC_CM AG08H and hIPSC_CM AG161B as well as the Costello CM_Cos HRAS p.G12A and CM_Cos HRAS p.G12S were derived from primary fibroblasts. All hiPSCs clones were tested for (**A**) stemness and (**B**) pluripotency markers expression using RT_QPCR based standardized procedures. The differentiation capacity was also tested in embryoid bodies collected at Day 14 post differentiation and RT qPCR analysis.

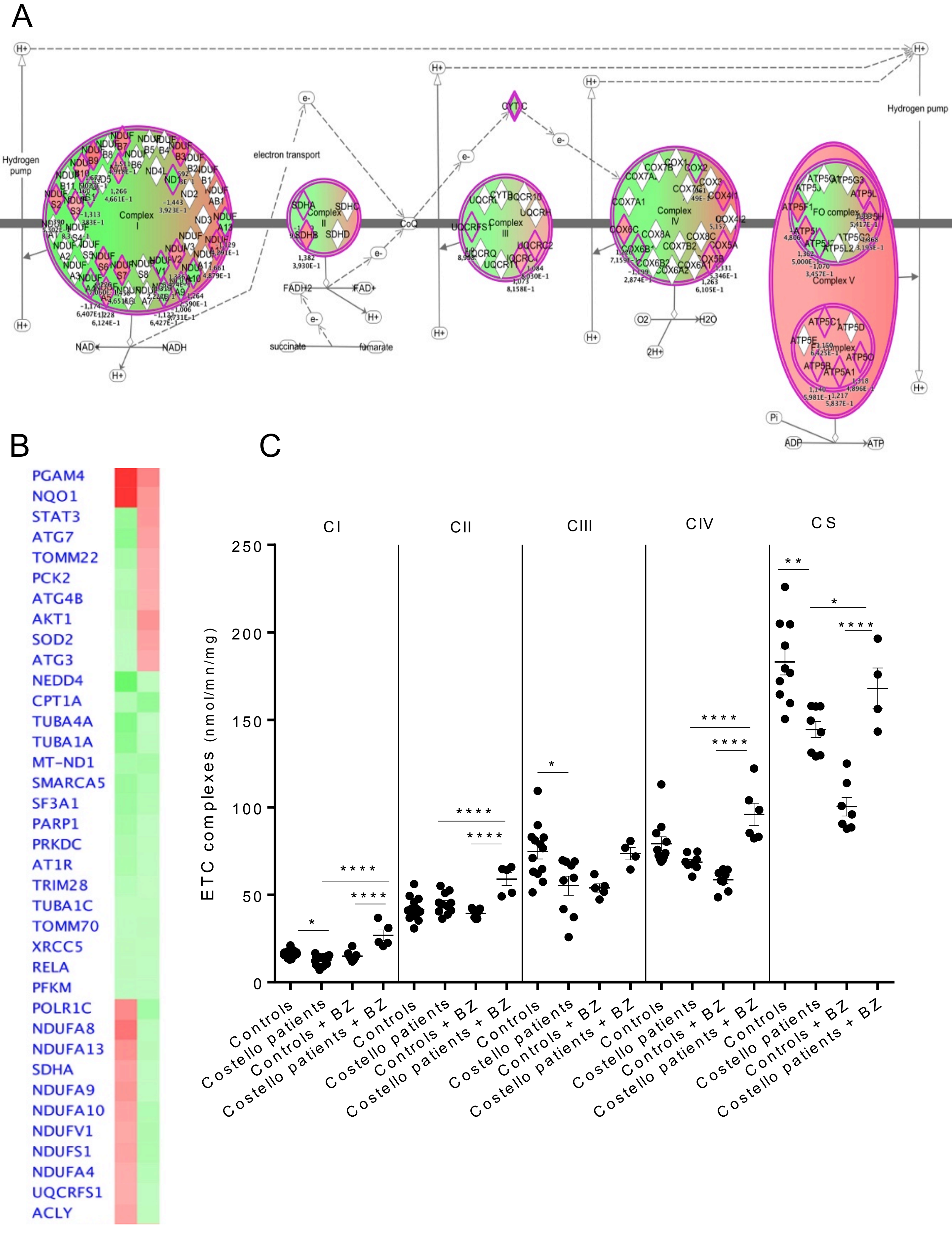

Figure S13: **Rescue of mitochondrial proteostasis in CS patient's skin fibroblasts treated during 48h with 500µM Bezafibrate.** (**A-B**) Data from the proteomic analysis of BZ treated cells compared to untreated (red = upregulated, green = down-regulated). (**C**) Respiratory chain complexes activities (n=3). The data were expressed as the mean ± SEM and compared using Anova with Dunett's correction.
*p < 0.05, **p < 0.01, ****p < 0.0001 (multiple t-test)

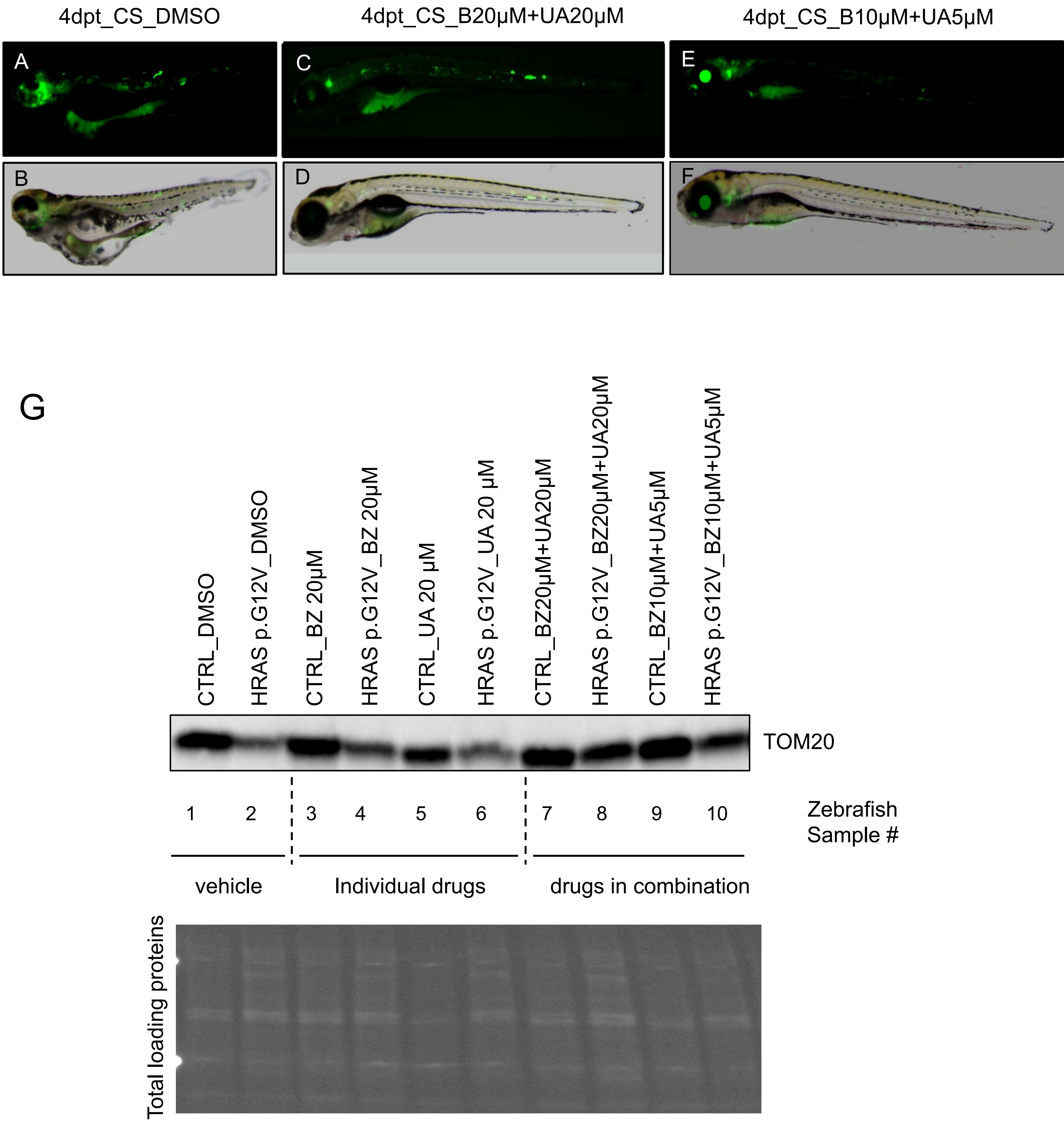

Figure S14: **CS Zebrafish model molecular characterization and treatment with the bezafibrate-urolithin A combination. A)** Observation of the expression of the HRASV12 plasmid in embryos at the 5 days post-fertilization stage and after 4 days of treatment using GFP. Embryos showing no defects after a combined treatment with bezafibrate and urolithin A at 20µM **(C-D)** or 10µM and 5µM respectively **(E-F)** express the plasmid HRASV12:GFP at the same level as the embryos treated with DMSO. **B)** Measurement of TOM20 (mitochondrial protein marker of organelle content) was performed by westerblot on whole zebrafish. Injected control or Costello animals were used and denominated as CTRL or HRAS p.G12V respectively. The zebrafish were treated with bezafibrate (BZ) or urolithin A (UA), alone or in combination. TOM20 expression was normalized to the total protein content in the different samples.

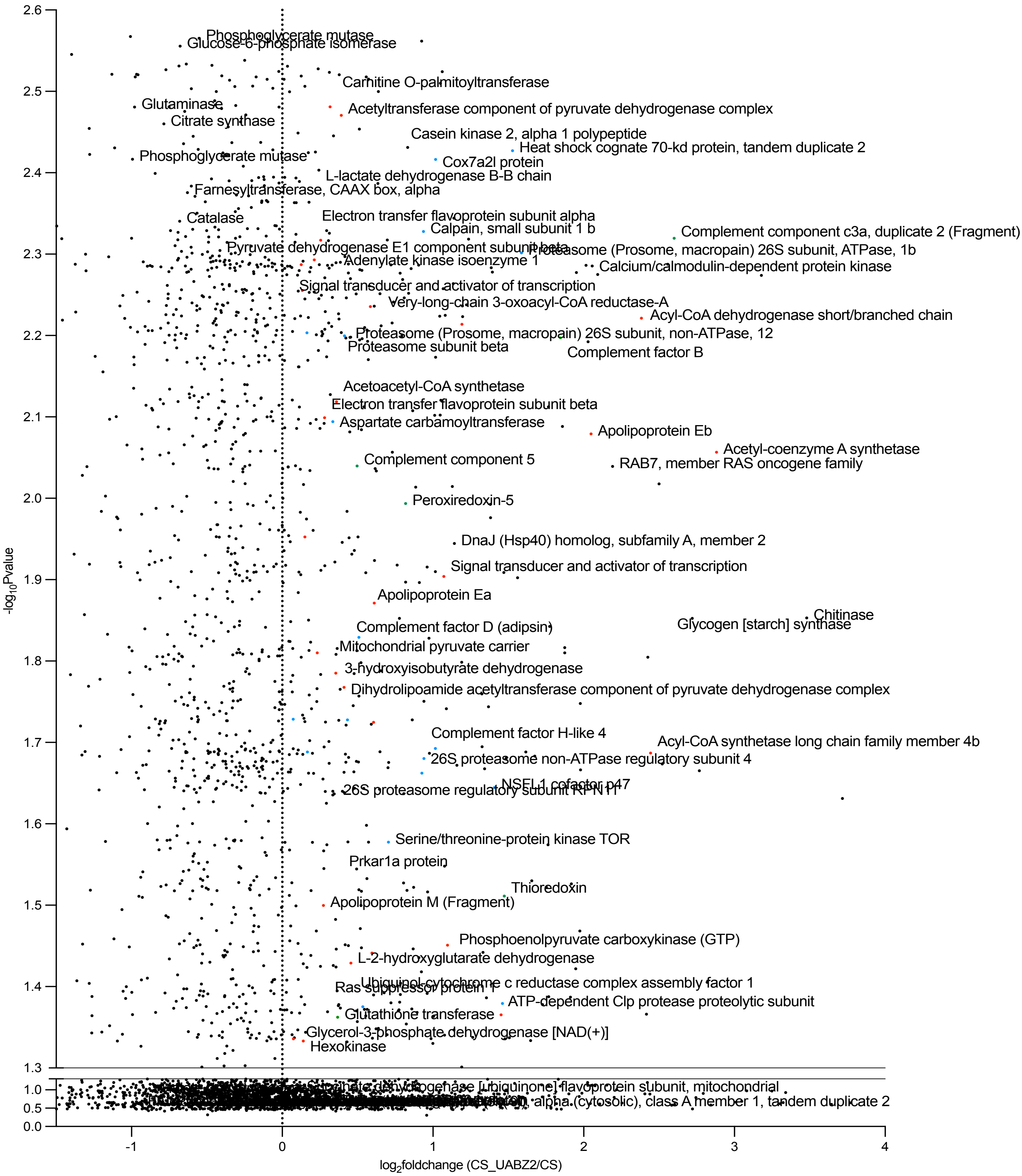

Figure S15: **Bezafibrate + Urolithin A combination mode of action (*in vivo*) analysis by untargeted proteomics.** Whole Zebrafish treated with BZ 20 µM + UA 5µM were used to obtain a protein lysate and to perform a label free proteomic analysis. The data of the differential proteome were shown as a Volcano plot. The $\log_2$fold change for each protein is shown in the abcissa (treated/untreated) and the $-\log_{10}$pvalue is shown on the Y axis. The significancy threshold was set at −logpvalue > 1.3.

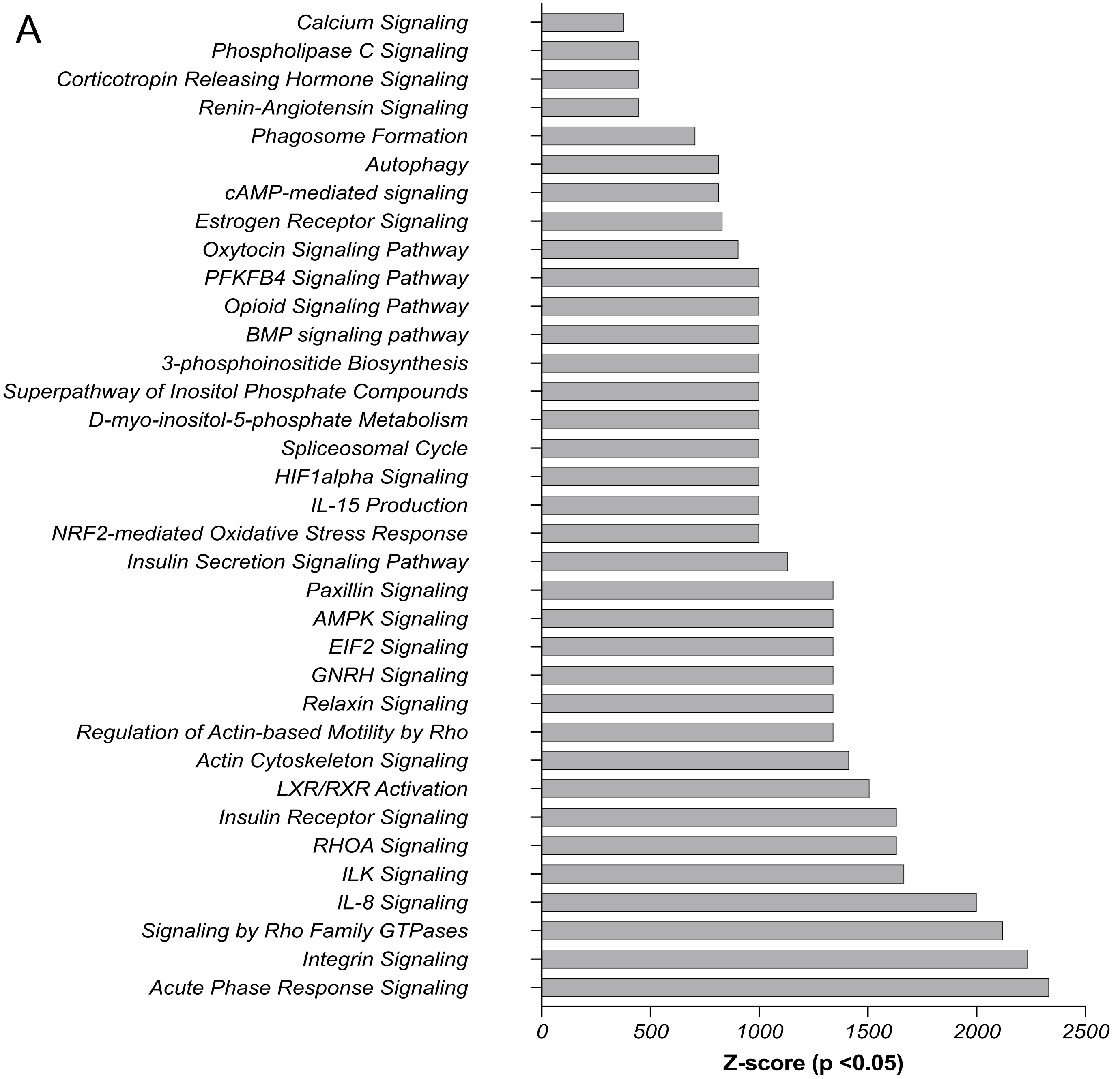

B

Symbol	Entrez Gene Name	UniProt Accession	Expr Fold Change	Type(s)
A2M	alpha-2-macroglobulin	A0A2R8RLB3	1000	transporter
AMBP	alpha-1-microglobulinbikunin precursor	A7E2Q2	1000	transporter
C3	complement C3	A0A0R4IMB2	1000	peptidase
AGT	angiotensinogen	Q8JH29	4.375	growth factor
SERPINA1	serpin family A member 1	Q5SPJ4	3.562	other
MAP2K4	mitogen-activated protein kinase kinase 4	B1NA68	3.009	kinase
CFB	complement factor B	F1R886	2.848	peptidase
SERPINF2	serpin family F member 2	F1RAP6	2.374	other
RBP4	retinol binding protein 4	Q9PT95	2.313	other
C5	complement C5	E7FCV2	1.625	cytokine
CP	ceruloplasmin	Q6P3G1	1.592	enzyme

Figure S16: **Bezafibrate + UrolithinA combination mode of action analysis using pathway analysis.** The data of the proteomic analysis (figure S15) were analyzed using IPA-Qiagen to identify the signaling and the metabolic pathways positively activated by the treatment. The top-score activated pathways are shown in **(A)**. The pathway with the highest score was 'Acute Phase Response Signaling' and the components of this pathway stimulated by the BZ+UA treatment in whole zebrafish were listed in **(B)**.

Methods.

1/ Construction of the mouse, cell and zebrafish models of the Costello Syndrome

1.1 Sampling and culture of Costello (CS) patient's skin fibroblasts. Primary skin fibroblasts were collected from the arm of Costello pediatric patients with *HRAS* mutation (p.G12A and p.G12S) at the genetic department of Bordeaux University Hospital. Patient's characteristics are given in **Table S1**. Skin fibroblasts from age-matched control subjects were also obtained from circumcision at Bordeaux University Hospital. All patients gave written informed consent to participate in this study, according to the Committee for the Protection of Persons (IDRCB N°: 2015-A00705-44, CPP 2014/44, Clinical Trial NCT02812511). The cells were then grown in Dulbecco's Modified Eagle Media (DMEM, Gibco) containing 5 mM glucose and supplemented with 5% fetal bovine serum (Gibco), 100 U/ml penicillin and 100 U/ml streptomycin (Gibco), in 5% CO_2 at 37 °C. For all experiments, the cells were harvested during the exponential phase of growth at 70% confluency. The primary fibroblasts cell lines were generated on the same day and the cells underwent similar number of passages at the moment of the various experiments.

1.2 Generation of HRAS p.G12A and HRAS p.G12S mutant human skin fibroblasts. The mEGFP-HRas plasmid (#18662, addgene) was mutated in HRAS p.G12A and HRAS p.G12S using QuikChange II Site-Directed Mutagenesis Kit (Agilent) according to the manufacturer's instructions. Subsequently, two lentiviral vectors were constructed by inserting HRAS p.G12A and HRAS p.G12S cDNA into the pLenti vector which contains the puromycin resistance gene (OriGene). Lentiviral particles were produced by transient transfection of 293T cells using a calcium phosphate transfection technique. Viral titer was determined by assessing enhanced-green fluorescent protein (EGFP) expression by flow cytometry. 80 000 cells were incubated with viral supernatants (MOI 10) for 48 h at 37°C. The clones expressing *HRAS* mutant were obtained by sorting the cells using the GFP expression and following puromycin treatment.

1.3 Generation of the HRAS p.G12S Costello mouse model. The original Costello (CS) HRAS p.G12S mouse model reported in this manuscript was produced and phenotyped by the Institut Clinique de la Souris (ICS) and funded by Association Française des Syndromes de Costello et Cardio-Facio-Cutané. The CS transgenic mouse model was generated in the 129SvPas*C57BL/6N genetic background through homolog recombination of R1 embryonic stem cells (ES). These ES R1 cells were derived from a male blastocyst obtained from the two 129 lineages, namely 129X1/SvJ and 129S1/SV-$+^{p}+^{Tyr-c}$ Kitl $^{Sl-J}$/+. An IRES-LacZ cassette had initially been inserted at the 3' position of the *Hras* transgene obtained from (101). This genetic construction was recombined to obtain the knock-in of HRAS p.G12S in the ES cells. Solely the clones with a *Hras* mutation on the allele initially targeted by the IRES-LacZ cassette were selected. Then, two clones were microinjected in blastocystes of C57BL/6N genetic background. Transmission of the transgene in the germinal lineage was obtained after crossing the chimeras resulting from the microinjection. Phenotyping of the CS mouse model was performed at ICS using the animal phenotyping platform described at http://www.ics-mci.fr/en/departments/phenotyping/. A summary of the phenotyping data of the HRAS p.G12S CS mouse is given in Figure S2 and a comparison with two other Costello mouse models is also provided.

1.4 Generation of the CS-hiPSC and derived human cardiomyocytes. The control AG08H hiPSC clone was derived from primary fibroblasts reference AG08498. The control AG161B hiPSC clone was derived from primary fibroblast reference AG162102. Both primary fibroblasts cultures were obtained from the Coriell Institute. The Costello p.G12S clone was derived from primary fibroblasts derived from a skin biopsy obtained from a female patient at age 10. The Costello p.G12A clone was derived from primary fibroblasts derived from a skin biopsy obtained from a male patient at age 5. All individuals have provided written informed consent for the collection of samples and subsequent analysis for medical research. The study was done in accordance with the Declaration of Helsinki. The study was deposited on Clinical Trial.gov with the reference: NCT02812511. All hiPSCs clones were derived from primary fibroblasts after transfection of different vectors (pCXCLE-hOCT3/4-shp53-F (Addgene ref 27077), pCXLE-hSK (Addgene, ref 27078, encoding *SOX2* and *KLF4*), pCXLE-hUL (Addgene,ref 27080, encoding *L-Myc* and *LIN28*) by Amaxa electroporation (NAPD 1001). Human iPS (hiPSCs) colonies were picked 4 to 6 weeks after transfection based on their ES cell-like morphology. Colonies were grown and expanded in mTeSR™1 medium (Stem cells) on BD Matrigel™ (BD Biosciences, cat, No. 354277) coated dishes. For each cell line 2 to 3 clones were fully characterized using standardized procedures (102) such as absence of expression of the reprogramming transgenes, expression of pluripotency markers by RT-qPCR, phosphatase alkaline staining and karyotype for checking the absence of chromosomal abnormalities. Expression of the Keratin Sulfate antigens Tra1-60 and Tra1-81 and the glycolipid antigen SSEA4 was verified by flow cytometry using the human pluripotent stem cell transcription factor analysis kit, (BD Biosciences, Ref 560589). Differentiation capacity was also tested in embryoid bodies collected at Day 14 post differentiation and RT qPCR analysis.The RNA extraction and quality control for hiPSC cells characterization was performed as follow. Total RNA was extracted using the RNAeasy kit (Qiagen) following manufacturer's instructions. Quality, quantification and sizing of total RNA was evaluated using the RNA 6000 Pico assay (Agilent Technologies Ref. 5067-1513) on an Agilent 2100 Bioanalyzer system. For Quantitative RT-PCR, Reverse transcription of 1 µg of total RNA was performed using the Superscript II kit and oligo dT following manufacturer's instructions at 42°C for 50 minutes followed by inactivation at 70°C for 15 minutes (Life Technologies). Primers were previously described (103). Real-time PCR amplification was performed on a LightCycler 480 (Roche) using the SYBR green master mix. All PCR were performed using a standardized protocol and data were analyzed with the Lightcycler 480 software version 1.5.0.39 (Roche). For each sample, fold-change was obtained by comparative quantification and normalization to expression of the *36B4* housekeeping gene used as standard for hiPS cells characterization. Data are expressed as means ± SEM.

Cardiac differentiation was performed using the StemCell cardiomyocyte differentiation (Ref 05010) and maintenance kits (Ref 05020). Briefly, after dissociation, single cell hiPSCs were seeded on pre-coated Matrigel plates in mTeSR supplemented with 10 µM Y-27632 to increase cell survival. At 95% confluency, cells are grown in StemDiff cardiomyocyte differentiation medium. Medium is sequentially replaced every two days following the manufacturer's instructions up to the 8th day post differentiation. At day 8, small beating areas might become visible and increase with time in culture. Between day 10 and 14, differentiation medium is replaced and cells are grown in the maintenance medium until day 30 with medium replacement every two days. The Flow cytometry analysis was performed as follow. Cells are treated with accutase at 37°C during 10 minutes and rinsed with cell medium. For each condition, 1×10^5 cells were fixed in paraformaldehyde 4% for 20 minutes. After spinning at 1000rpm for 5 min, cells were rinsed with BSA 0.5 %, treated with Triton 0.2% for 20 mins and blocked with BSA 0.5% for 20 min prior to the addition of the different antibodies (5 µl of antibody in 100 µl of BSA 0.5%). Samples were incubated for 30 min. Samples were then spin down and rinsed and analyzed using an ACCURI C6 flow cytometer. Antibodies were purchased from BD Biosciences (anti-TRA1-60; anti-TRA1-81, anti-SSEA4, human pluripotent stem cell transcription factor analysis kit, Ref 560589). For cardiac cells characterization, 10^6 cells were collected after dissociation using the Stemcell dissociation reagent (ref 05025). Cells were fixed with 300 µl CytoFix/CyotPerm (BD

Biosciences) and permeabilized using the Perm/Wash solution. Cells were stained using the Anti Troponin T antibody coupled with FITC (Ref 130-106-745, Milteny Biotech) or the REA control isotyope coupled to FITC (Ref 130-104-611, Milteny Biotech). Cells were analyzed on the basis of Forward Scatter (FSC-A) versus side scatter (SSC-A) for selection of live cells and elimination of cell aggregates or debris. Analysis of fluorescent population was limited to live cells. Unstained cells and isotype controls were used to determine the background of fluorescence and compensation was determined for individual fluorochromes. For determination of the percentage of Troponin T positive cells, cardiomyocytes were collected at day 30 post differentiation and treated as indicated. We obtained 72.55% ± 12% of Troponin-positive cells for the AG08H control clone, 55.88% ± 15.6% for the AG161 control clone, 77.89% ± 2.3% for the Costello G12A clone and 86.75% ± 8.7%.

1.5 Generation, treatment and analysis of the Zebrafish model of the Costello syndrome. Zebrafish (Danio rerio) embryos were obtained from natural spawnings and raised in embryo medium (E3) (104) at 28.5°C. Embryos were staged as described previously (105). Starting 5 dpf, embryos were fed daily. All the studies conducted for this manuscript have been reviewed and approved (F341725 / 21063-2019061317218694). The GFP-H-RASV12 plasmid was kindly provided by the Dr. Marina Mione from the Department of Cellular, Computational and Integrative Biology (CIBIO) - University of Trento and used as described in (54). 1 nl of 12.5ng/µl of GFP-H-RASV12 plasmid and 12.5ng/µl of T2 transposase mRNA were co-injected in the first cell of zebrafish eggs. Control embryos were injected with the same volume of PBS. H-RASV12 was expressed in zebrafish embryo under heat shock, 30mn at 37°C at 1day post fertilization (dpf) and embryo expressing GFP at 2 dpf were selected for the study.

The embryo injected with the HRASV12 plasmid were treated daily by bathing from 1dpf to 5dpf with respectively 20µM bezafibrate, 20µM urolithine A, 20µM bezafibrate + 20µM urolithine A, 10µM bezafibrate + 5µM urolithine A, 5µM bezafibrate + 5µM urolithine A. Control embryos were treated with the same volume of DMSO corresponding to 0.2% of DMSO. The imaging and phenotype analysis of mortality and defects of appearance after plasmid injection were performed on alive embryo anesthetized using ethyl 3-aminobenzoate methanesulfonate, MS-222 (Sigma, Cat.# A-5040) at 200 mg/L at 2 days post fertilization, 3 dpf, 4 dpf and 5 dpf using a ZEISS axiocam stereomicroscope (ZEISS, Germany).

2/ Biochemistry and molecular biology methods

2.1 HRAS pull-down activity assay. HRAS activity status was evaluated using the H-Ras Activation Assay Kit (Abcam, #ab211158) according to the manufacturer’s instructions.

2.2 Protein extracts, proteomic analysis and western blots. Fibroblasts cells pellets were lysed using a lysis buffer containing RIPA 1X buffer (Sigma-Aldrich, #R0278) supplemented with 1:100 Phosphatase inhibitor (Roche #04906845001) and 1:100 Protease inhibitor cocktail (chymostatin, leupeptin, antipain, pepstatin A – Sigma-Aldrich). Protein lysates were placed on ice for 15 min, vortexed and cleared by centrifugation at 12,000 × g for 15 min at 4 °C. The supernatants were retrieved and frozen at −80 °C until use. Protein concentration was evaluated using the BCA Protein Assay kit – Reducing Agent Compatible according to the manufacturer’s instructions (Thermo Fisher Scientific # 23250). Immediately after tissues removal, tissues were snap frozen in liquid nitrogen and stored at -80°C. Tissue lysis was performed in potter using RIPA 1X buffer (Sigma-Aldrich) supplemented with 1:100 Phosphatase inhibitor (Roche #04906845001) and 1:100 Protease inhibitor cocktail (chymostatin, leupeptin, antipain, pepstatin A – Sigma-Aldrich). The lysate was centrifuged for 20 min at 12,000 rpm at 4°C and the supernatant was used for the proteomic analysis and western blots.

Label-free quantitative proteomics: this analysis was performed by the proteomics core facility at University of Bordeaux (https://proteome.cgfb.u-bordeaux.fr/en). The steps of sample preparation, protein digestion and nano-liquid chromatography–tandem mass spectrometry analysis were performed as previously described (106). For protein identification, Sequest HT and Mascot 2.5 algorithms through Proteome Discoverer 1.4 Software (Thermo Fisher Scientific Inc.) were used in batch mode by searching against a Homo sapiens database (70 671 entries, Reference Proteome Set, release 2017_05) or a merge of protein databases: Homo sapiens and Mus musculus (121 170 entries, Reference Proteome Set, release 2017_05). Databases were downloaded from http://www.uniprot.org/ website. Two missed enzyme cleavages were allowed. Mass tolerances in MS and MS/MS were set to 10 ppm and 0.02 Da. Oxidation of methionine, acetylation of lysine and deamidation of asparagine and glutamine were searched as dynamic modifications. Carbamidomethylation on cysteine was searched as static modification. Peptide validation was performed using Percolator algorithm (107) and only "high confidence" peptides were retained corresponding to a 1 % False Positive Rate at peptide level. Raw LC-MS/MS data were imported in Progenesis QI (version 2.0; Nonlinear Dynamics, a Waters Company) for feature detection, alignment, and quantification. All sample features were aligned according to retention times by manually inserting up to fifty landmarks followed by automatic alignment to maximally overlay all the two-dimensional (m/z and retention time) feature maps. Singly charged ions and ions with higher charge states than six were excluded from analysis. All remaining features were used to calculate a normalization factor for each sample that corrects for experimental variation. Peptide identifications (with FDR<1 %, see above) were imported into Progenesis. A statistical criteria of ANOVA p-value less than 0.05, a minimum of two peptides matched to a protein, and a ≥2- fold change in relative abundance between the two conditions (n= 3 in each group) were used as the criteria for identification as a differentially expressed protein. Noticeably, only non-conflicting features and unique peptides were considered for calculation at protein level. Proteins were clusterized according to their functions by using the Kyoto Encyclopedia of genes and genome analysis in the search tool for retrieval of interaction between genes and proteins (STRING) database. More global analysis of the data was performed via use of Ingenuity Pathway Analysis (Qiagen).

Western blots: 20 µg protein extracted were dissolved in Laemmli buffer (Bio-Rad, #1610747) containing 4% β-mercaptoethanol (Sigma, #M3148) by incubation for 5 min at 95°C and loaded onto a 4-15 % SDS-page gel (BioRad, 4561083). Proteins were transferred electrophoretically onto PVDF transfer membrane (BioRad, # 1704157) for 7 min at 2.5 Amp in Transfert buffer with Trans-Blot Turbo (BioRad). Membranes were blocked 2h in BSA (Sigma Aldrich, # A2153) and incubated overnight at 4°C with the appropriate primary commercial antibodies: β-actin (1:10,000; Sigma #A5441), AMPKα (1:1000, Cell Signaling, #2532), Phospho-AMPKα (Thr172) (1:1000, Cell signaling, #2535), PGC1α (1:1000, ProteinTech, #66369), P-p70S6K (Ser371) (1:1000, Cell signaling, # #9208), LC3 (1:1000, Cell Signaling, #4108), TFAM (1:1000, Cell signaling, #8076S), ACC (1:1000, Cell signaling #3676S), P-ACCser79 (1:1000, Cell signaling #11818S), SQSTM1/p62 (1:1000, Cell signaling #39749S), LKB1 (1:1000, Cell signaling #3047S), P-LKB1(S428) (1:1000, Abcam #Ab63473), CRIF1 (1:1000, Abcam #226244), Parkin (1:1000, Cell signaling #2132S) and SPG7/PGN (1:1000, Abcam #Ab96123). Specific binding of antibodies was detected using appropriate peroxidase-conjugated secondary antibodies (1:5000, Biorad, #170-6516, #170-6515) or fluorescence antibodies (1:5000, Li-Cor Bioscience, #926-68070, #926-32211), and was visualized by enhanced chemiluminescence detection (Biorad) or Fluorescence

detection (Li-Cor Bioscience, Odyssey) respectively. Densitometry analyses of immunoblots were performed using ImageJ software.

3/ RNA extraction, DNA synthesis and real time PCR (qPCR). RNAs were extracted from cells using Qiagen RNeasy kit (Qiagen, #74104) following the manufacturer's recommendations. Total RNA was quantified spectrophotometrically and RNA quality was verified using Agilent RNA 6000 Nano kit on an Agilent 2100 Bioanalyzer (Agilent Technologies). cDNA synthesis was performed using iScript cDNA Synthesis kit (Bio-Rad, #1708890). Gene expression analysis was performed using CFX96™ Real Time PCR (Bio-Rad) and iQ SYBR Green supermix (BioRad, #1708882). Primer pairs were purcheased by pre-desigers primers from Sigma-Aldrich (KiCqStart® SYBR® Green Primers Predesigned primers for gene expression analysis). The comparative Ct method was used to compare changes in gene expression levels. *GUSB* and *ACTB* were used as an endogenous control.

4/ miRNA extraction, cDNA synthesis and real time PCR (qPCR). miRNAs were extracted from cells using mirVana™ miRNA Isolation Kit (Thermo Fisher Scientific, #AM1560) following the manufacturer's recommendations. miRNAs was quantified using Agilent smallRNA 6000 Nano kit on an Agilent 2100 Bioanalyzer (Agilent Technologies). cDNA synthesis was performed using miScript II RT Kit (Qiagen, #218161). Gene expression analysis was performed using CFX96™ Real Time PCR (Bio-Rad) and miScript SYBR® Green PCR Kit (Qiagen, # 218076). Primer miRNA were purcheased from Qiagen. The comparative Ct method was used to compare changes in gene expression levels. Hsa-miR-30e and hsa-miR-16-2 (Qiagen, #MS00009401, # MS00008813) were used as an endogenous control.

5/ Cell transfections. Cell transfection was carried out according to the manufacturer's recommendations from HiPerFect Transfection Reagent kit (Qiagen, #301705), using 10 nM of the following small RNAs: hsa-miR-221-5p miRCURY LNA miRNA Inhibitor and Negative Control B (Qiagen, # YI00199007).

6/ Bioenergetic investigations in CS mouse heart and CS human skin fibroblasts.

6.1 Polarography: To assay intact skin fibroblasts cell respiration we used high-resolution respirometry (Oroboros O_2k-system). For each run 0.5 million cells per milliliter of cell culture medium was used. Three technical replicates were performed for three different cel cultures (biological replicates; n = 3). The rates of respiration were normalized per million of cells. The term 'routine' respiration is defined as the respiratory rate of intact cells measured in 5 mM glucose DMEM (Gibco) under atmospheric conditions at 37 °C. The term 'leak' respiration is the respiratory rate measured in the routine conditions after addition of the F_1F_O-ATPsynthase inhibitor oligomycin A at 5µM (Sigma-Aldrich, #75351). This 'oligo' state of respiration does not depend on ADP phosphorylation. The term 'CCCP' respiration defines the rate of respiration measured in the 'oligo' conditions after addition of the uncoupler CCCP (Sigma, #C2759) used until 1 µM. The 'CCCP' state allows to evaluate the maximal capacity of the respiratory chain in presence of energy substrates and oxygen concentration as defined in the 'routine' conditions. The 'routine/oligo' ratio allows to quantify the coupling degree of the respiratory chain with ADP phosphorylation in the routine conditions (Sigma, # A5285). The FCCP/Routine ratio gives a measure of the capacity of the respiratory chain to be chemically uncoupled. It indicates how far from the maximal capacity the routine respiration operates.

6.2 Permeabilization and respiration analysis of CS mouse muscle fibers. Permeabilization of the skeletal muscle fibers was obtained as follow: bundles of fibers between 10 and 20 mg were incubated for 15 minutes in 5 mL of solution A (EGTA 10 mM, $MgCl_2$ 5 mM, taurine 20 mM, DTT 0.5 mM, imidazole 20 mM, K^+MES 0.1 M, pH 7.0, ATP 5 mM, and phosphocreatine 20 mM) containing saponin 50 µg/mL. The bundles were then washed three times for 5 minutes each time in solution B (EGTA 10 mM, $MgCl_2$ 5 mM, taurine 20 mM, DTT 0.5 mM, imidazole 20 mM, K^+MES 0.1 M, pH 7.0, KH_2PO_4 3 mM, and 5 mg/mL fatty acid-free BSA) to remove saponin. All procedures were carried out at 4°C with extensive stirring. The oxygen consumption rate was then measured polarographically at 37°C using a Clark electrode (Hansatech Instruments Ltd., Norfolk, United Kingdom) connected to a computer that gave an on-line display of rate values. The respiratory rates were determined in an oxygraph cuvette containing one bundle in 1 mL of solution B. To express the respiration rates per mg of fibers after a respiration run, the fiber is dried and weighed. The respiratory activities are expressed in O/min/mg of fiber. Fiber respiration was determined under routine condition (pyruvate 10 mM and malate 9 mM), or in ADP state (1mM) or in presence of inhibitor cocktail (0.3 µM rotenon, 100 µM Antimycin A and 7µM KCN).

6.3 Cellular and mitochondrial ATP synthesis: the intracellular ATP content of human skin fibroblasts was measured using the bioluminescent CellTiter-Glo 2.0 Assay (Promega, #G9241). 30 000 cells were plated in a 96-well plate. Cells were treated or not with inhibitor coktail during 30 minutes (Antimycin A 100µM (Sigma, #A8674), Rotenone 0.5µM (Sigma, #R887) and oligomycin 3µM (Sigma-Aldrich, #75351) and lysed using the lysis buffer provided with the kit and the lysates were protected from light. ATP concentration was determined by the light-emitting luciferase-catalyzed oxidation of luciferin with ATP and bioluminescence measurement on a luminometer (Labsystems Luminoskan). Standardization was performed using known quantities of standard ATP. Mitochondrial ATP corresponds to total ATP content minus ATP content under inhibitory cocktail.

6.4 Mitochondrial fibers ATP synthesis rate: Muscle fibers were permeabilized in solution A, as described above, and placed in buffer containing EDTA 20µM, iodoacetate 1mM, ap5A 50µM, pyruvate 10mM and malate 9mM. The basal ATP production were measured every 30 seconds during 2 minutes, 10µL were taken. ADP at 1mM was added in buffer and ATP production were measured every 10 seconds during 1 minute. To inhibit mitochondrial ATP production, a cocktail inhibitor was added (Antimycin 100µM (Sigma, #A8674), Rotenone 10µM (Sigma, #R887), Oligomycine 10µM (Sigma-Aldrich, #75351)) and ATP production was measured every 30 seconds during 2 minutes. The reaction was stopped by adding 100 µL of DMSO, and 5 mL of water. The intracellular ATP content was measured using the bioluminescent CellTiter-Glo 2.0 Assay (Promega, #G9241) and the ATP production was normalized to the mg of fiber.

6.5 Mitochondrial electric transmembrane potential: Determination of the mitochondrial transmembrane electric potential was performed by fluorescence imaging using tetramethylrhodamine methyl ester (Invitrogen, #I34361). Briefly, cells were incubated with 250 nM TMRM during 30 minutes at 37 °C and fluorescence detection was performed on a SAFAS Xenius.

6.6 Measurement of the enzymatic activities of respiratory chain complexes and citrate synthase: We determined several respiratory chain complexes and citrate synthase enzymatic activities, as described in (108) and detailed after :

1. Complex I (NADH ubiquinone oxidoreductase): each mice tissue was incubated at 37°C, with 100 µM decylubiquinone, 50 mM potassium phosphate (pH 7,5) and 3,75 mg/mL BSA. Reaction was started by the addition of 100 µM NADH. In patients fibroblasts, sample was incubated at 37°C, with 200 µM decylubiquinone, 50 mM potassium phosphate (pH 7,5) and 3,75 mg/mL BSA. NADH absorbance decrease was followed at 340 nm during 3 min, either in presence or in absence of 12,5 µM of rotenone, a specific complex I inhibitor. The difference between these 2 activities, gave the rotenone sensitive activity, which correspond to the specific activity of respiratory chain complex I.
2. Complex II (succinate ubiquinone oxido-reductase) in mice tissue and patients fibroblasts: each sample was incubated at 37°C, with, 25 mM K Phosphate pH 7.5, 20 mM succinate, 50 µM 2,6- dichlorophenolindophenol (DCPIP), 1 mM KCN, 100 µM ATP, 2 mg/mL BSA. Reaction was started by adding 100 µM decylubiquinone. DCIP reduction rate was measured at 660 nm during 5 min.
3. Complex III (ubiquinol cytochrome c oxidoreductase) in mice tissue and patients fibroblasts : each sample was incubated at 37°C, with 50 µM cytochrome c, 100 mM potassium phosphate (pH 7,5) , 250 µM EDTA and 2.5 mg/mL BSA. The reaction was started by the addition of 200µM decylubiquinol. Cytochrome c reduction rate was measured at 550 nm during 3 min, with and without 12,5 µg/ml antimycin A, a specific complex III inhibitor. The difference between these 2 activities, gave the specific activity of respiratory chain complex III. Decylubiquinol was obtained as described: add a few crystals of potassium borohydrite in 600 µl of a 25 mM decylubiquinone solution with gentle mixing until the solution becomes colorless. Then, add 21 µl of 3M HCl, centrifuge at 12000 RPM for 5 min, 4°C. The supernatant was decanted into a fresh tube and these operations (HCl addition and centrifugation) were repeated and finally decanted in fresh tube.
4. Complex IV (cytochrome c oxidase) : In mice tissue, assay medium was composed of 50 mM potassium phosphate (pH 7) and 100 µM cytochrome c reduced to 95%. In patients fibroblasts, assay medium was composed of 50 mM potassium phosphate (pH 7) and 50 µM cytochrome c reduced to 95%. It was incubated at 37°C. Reaction was triggered by the addition of the sample. The cytochrome c oxidation rate was measured at 550 nm during 5 min.
5. Citrate synthase (CS) : each sample was incubated at 37°C, with 300 µM acetylCoA, 100 µM DTNB, 100 mM TrisHCl (pH 8.1) and 0,1% triton. The reaction was started by the addition of 10 mM oxaloacetic acid. Increase of TNB absorbance was followed at 412 nm during 4 min.

7/ Imaging methods

7.1 Immunofluorescence. Cells were seeded into Labteck II (Chamber Slide™ System 8 chambres, fisher Scientific) at 5000 cells per chamber. Mitotracker Red (Invitrogen, #M22425) was incubated at 50nM during 10 minutes in 5% CO_2 at 37 °C. Then, briefly, cells were fixed with 4 % paraformaldehyde 20 minutes. Cells were rinsed twice with PBS and DAPI was added during 5 minutes. Cells were finally rinsed twice with PBS and Fresh PBS were added to chamber to maintain cells integrity. Pictures were taken in epifluorescence

microscope (ZEISS, objective x20) and mitochondrial network was analyzed using Morphostryder (Explora Nova) Software.

7.2 COX/SDH histochemistry. Frozen muscle sections (10 µm) were dried and incubated with COX solution for 2 hours at room temperature. Following a PBS wash the sections were overlaid with SDH solution and incubated for 1 hour at 37 °C. The number of COX+ and COX- fibers was counted using ImageJ software to do statistical analyses.

7.3 Hemalun and eosin staining. Muscle tissues taken from mice were snap frozen in cold isopentane and 10 µm sections were cut in cryostat. The sections were stained with HE using standard procedures for proper muscle orientation and morphological assessment. Images were taken using optic microscope (ZEISS, objective x20) and the cross section of LV, RV and the diameter of the mice heart were measured using ImageJ software.

8/ Preclinical study

8.1 Bezafibrate (BZ) treatment *in vitro.* For BZ treatment, cells were incubated in fresh media containing 500 µM bezafibrate (Sigma, #B7273) or vehicle only (0.01 % DMSO) for 48 h.

8.2 Preclinical study in CS mouse

All experiments were performed in accordance with the European guidelines for animal experimentation at the Institut Clinique de la Souris (ICS). First, animals were acclimated for 1 week in a phenotyping area with controlled temperature and access to food following a 12/12 day/night cycle with food and water *ad libitum*. At 5 weeks of age and until 21 weeks of age the animals received standard diet enriched with 0.05% bezafibrate based on previous studies (50). Once a week systolic blood pressure was measured as well as heart beating rate. At necropsy the organs were weighted.

9/ Echocardiography

Transthoracic Echocardiographic images were captured using a VisualSonics Vevo 2100 Imaging System (Toronto, Canada) with a MS400 probe (30-MHz). The echographers allow M-mode, B-mode, and Doppler imaging with ECG and respiratory gating of all images. The thorax was treated by mechanic shaving and hypoallergenic cream hair remover (Nair^{TM}) to optimize the acoustic interface. Mice were anesthetized using 1–2% isoflurane in oxygen and positioned supine on the heating pad of the Vevo system, which maintains normo thermia by continuous monitoring of the rectal temperature. Prewarmed ultrasound gel was applied on the thorax. The VisualSonics rail system was used to fix the probe, avoiding any compression of the thorax. The cardiac morphology and ventricular systolic function were acquired by M-mode tracings of the LV using the short-axis view, with the ultrasound beam perpendicular to the LV at the midpapillary level to determine ejection fraction (EF), fractional shortening, wall thickness, LV inner diameter (LVID), and LV mass (LVM= 1.055x [(EDD+SW+PW) 3-EDD3)]). Aortic dimensions were determined using B-mode imaging in the parasternal long-axis views. Pulmonary and aortic artery velocity and pressures to detect intra-cardiac pressures changes (Aov and RV function) was evaluated using color Doppler imaging in pulmonary artery and aortic arch.

The Diastolic function were assessed by color Doppler mode from the apical 4 chamber view for Mitral valve (MV) and tricuspid valve (TV) to detect left and right diastolic filling patterns. The MV measurements performed were the following: MV early wave peak (MVE), MV atrial wave peak (MVA), aortic ejection time (AET), isovolumic relaxation time (IVRT), isovolumic contraction time (IVCT). The TV measurements included TV early wave peak (TVE) and TV atrial wave peak (TVA). The TV peak pressure gradient (TVPPG) and TVE/A ratio were calculated

Images were captured on cine loops at the time of the study and afterward measurements were done off-line using Vevo Lab analysis software (version 3.3.5). The statistical analysis was obtained by Graph Pad (version 8.0.3).

10/ Statistics

All values are the mean ± SEM of minimal three independent experiments (biological replicates). The statistics were performed with GraphPad Prism 9 software using a 2-way unpaired Student's t test to compare two independent groups or paired for sequential measurements. Evaluation of the gaussian distribution of the data was performed prior to use t-test or ANOVA. One-way and two-way ANOVA with Dunnett's test correction were performed when comparing different groups (as precised in the legends). Statistical significance was determined at $^{*}P < 0.05$, $^{**}P < 0.01$, $^{***}P < 0.001$.
